# Dietary essential amino acids restore liver metabolism in ovariectomized mice *via* hepatic estrogen receptor α

**DOI:** 10.1038/s41467-021-27272-x

**Published:** 2021-11-25

**Authors:** Sara Della Torre, Valeria Benedusi, Giovanna Pepe, Clara Meda, Nicoletta Rizzi, Nina Henriette Uhlenhaut, Adriana Maggi

**Affiliations:** 1 Department of Pharmaceutical Sciences, University of Milan, Milan, Italy; 2 Center of Excellence on Neurodegenerative Diseases, University of Milan, Milan, Italy; 3 Department of Health Sciences, University of Milan, Milan, Italy; 4 Research Services Management Office, University of Milan, Milan, Italy; 5 Molecular Endocrinology, Institute for Diabetes and Cancer (IDC), Helmholz Zentrum Munich, Helmholtz Diabetes Center (HMGU), Munich, Germany; 6 Metabolic Programming, TUM School of Life Sciences Weihenstephan, Munich, Freising Germany

**Keywords:** Transcription, Transcriptomics

## Abstract

In female mammals, the cessation of ovarian functions is associated with significant metabolic alterations, weight gain, and increased susceptibility to a number of pathologies associated with ageing. The molecular mechanisms triggering these systemic events are unknown because most tissues are responsive to lowered circulating sex steroids. As it has been demonstrated that isoform alpha of the estrogen receptor (ERα) may be activated by both estrogens and amino acids, we test the metabolic effects of a diet enriched in specific amino acids in ovariectomized (OVX) mice. This diet is able to block the OVX-induced weight gain and fat deposition in the liver. The use of liver-specific ERα KO mice demonstrates that the hepatic ERα, through the control of liver lipid metabolism, has a key role in the systemic response to OVX. The study suggests that the liver ERα might be a valuable target for dietary treatments for the post-menopause.

## Abstract (web summary)

Menopause is associated with metabolic changes and an increased risk of a number of chronic diseases. Here, the authors show in a mouse model of menopause that an amino acid-enriched diet rescues liver lipid metabolism and prevents weight gain in a manner dependent on hepatic ERα.

## Introduction

In the entire course of phylogenesis, the liver, or its ancestral counterpart, has a primary role in the control of reproduction because responsible for the synthesis of the proteins indispensable for the maturation of the egg; in turn, the gonads regulate several hepatic metabolic functions associated with the reproductive needs. Such a tight, reciprocal control is strictly maintained throughout evolution from oviparous to viviparous species^1^. However, conceivably, in female mammals, the gonadal control of liver metabolism must have been perfected to enable this organ to recognize and respond to the increased number of solicitations associated with the dramatic metabolic changes necessary for the diverse reproductive phases (maturation of the follicle, pregnancy, lactation)^1–3^. This view is supported by the observation of a profound sexual dimorphism that characterizes this organ in mammals reported by several authors^2,4–7^.

Our previous studies showed that the main mammalian hepatic sensor of gonadal functions is the isoform alpha of the estrogen receptor (ERα). ERα regulates liver metabolism according to food availability and to the needs of the reproductive system, as exemplified by the demonstration that liver lipid metabolism is most sensitive to fasting^8–11^ and to circulating estrogens^12–15^. In addition, in the course of the reproductive cycle, high plasma content of estrogens is associated with the blockade of de novo lipid synthesis and changes in lipid transport, with increased clearance of LDL (low-density lipoproteins) and VLDL (very low-density lipoproteins), synthesis of HDL (high-density lipoproteins), changes of lipoprotein modifiers and liver receptors for lipoproteins^12,13^. All of these changes are abolished by the selective ablation of the hepatic ERα (LERKO)^12,13^.

To deploy this complexity of functions liver ERα must be subjected to a multiplicity of signaling molecules that enable this powerful transcription factor and its co-regulators to select and modulate a plethora of tissue-specific intracellular biochemical pathways^16^. In line with this reflexion, our laboratory showed that, in addition to estrogens, other molecules such as amino acids (AA) may activate the unliganded ERα^9^. This AA-dependent mechanism of ERα activation is necessary for the progression of the estrous cycle and represents an evolutionary well-conserved pathway to block fertility in case of dearth of nutrients^9^. Possibly as a consequence of the relevance of AA signaling in female liver, the metabolism of these nutrients is sexually dimorphic as demonstrated by studies carried out using different dietary regimens such as fasting^8^ or a diet enriched in fats (HFD)^17^. In case of short-term fasting, the female liver catabolizes the AA to fuel lipid synthesis by generating NADPH through the pentose phosphate pathway; this metabolic shift does not occur in LERKO females and in males^8^. In case of prolonged exposure to HFD, females conserve AA homeostasis and do not accumulate lipids in the liver; this is not the case for LERKO females and for males (independently of the presence or not of hepatic ERα), in the liver of which HFD induces significant lipid deposition and a decrease in the content of essential (EAA) and branched (BCAA) amino acids^17^. These experiments indicate that in female mice the hepatic ERα has a major role in preserving lipid and AA homeostasis.

Considering that in female mammals, including humans, the cessation of ovarian functions has severe cardiometabolic consequences^1,7,18–21^, and given the role of ERα in liver metabolism^12,13,22^ and the ability of AA to stimulate the activity of hepatic ERα in fertile females^9^, we hypothesized the potential of a diet enriched in EAA to counteract the metabolic derangements consequent to the decreased plasma estrogen levels. We, therefore, tested in ovariectomized (OVX) female mice the effects of a diet enriched in EAA and designed to retain the *ratio* among macro-nutrients.

The results obtained show that the *AA diet, by restoring liver metabolic homeostasis, offsets the OVX-induced lipid deposition in the liver and whole-body weight gain. This is achieved through the hepatic ERα. These findings are in line with studies showing correlations between the presence and severity of fatty liver/NAFLD (non-alcoholic fatty liver disease) and lowered hepatic content of BCAA^23,24^ and point to hepatic ERα as a valuable target for interventions to limit the dysmetabolism associated with women ageing and to the therapeutic potential of the *AA diet.

## Results

### A diet modified in the composition of EAA/BCAA counteracts the metabolic effects of ovariectomy

**Fig. 1:**
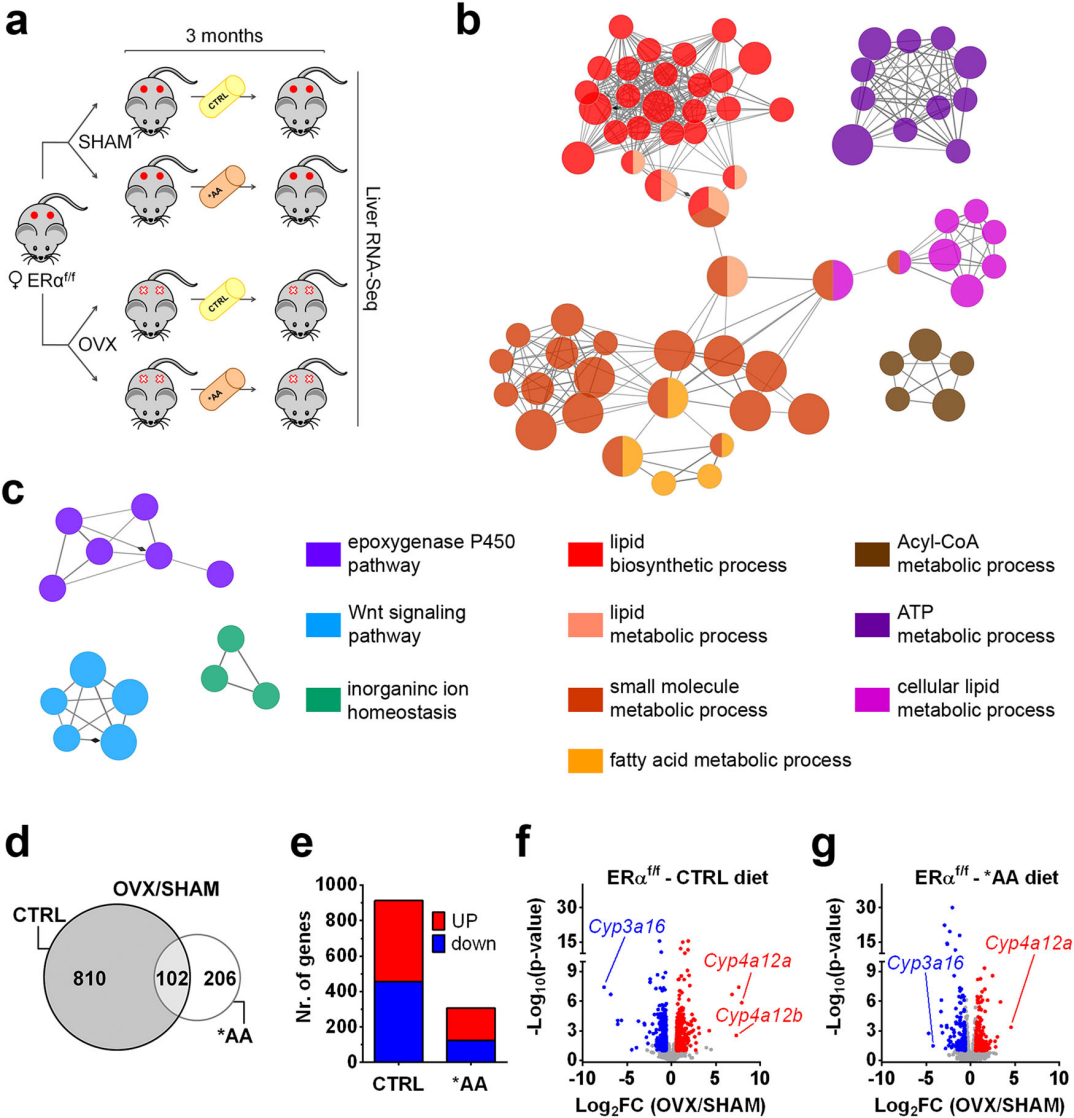
Dietary EAA/BCAA mitigate the differences in liver transcriptome between SHAM-operated and OVX females. **a** Experimental design adopted to evaluate the relevance of estrogens in the liver metabolic response to *AA diet. **b**–**c** Cluster analysis of the functional networks significantly up- (**b**) and downregulated (**c**) by OVX in the liver of ERα^f/f^ CTRL-fed females by RNA-Seq analysis (*n* = 4). **d** Venn diagram showing the genes differentially expressed in OVX *versus* SHAM mice. DEGs were identified by RNA-Seq (*n* = 4) in the liver of SHAM *versus* OVX ERα^f/f^ females fed with CTRL diet (grey circle, 912 genes) or *AA diet (white circle, 308 genes). **e** Distribution of genes up- and downregulated by OVX measured by RNA-Seq analysis (*n* = 4) in the liver of ERα^f/f^ females fed with CTRL or *AA diet. **f**–**g** Volcano plot of liver DEGs measured by RNA-Seq (*n* = 4) in SHAM and OVX ERα^f/f^ females fed with CTRL (**f**) or *AA (**g**) diet. OVX/SHAM *ratio* of gene expression is shown on the X axis as Log_2_FC and significance is displayed on the Y axis as −Log_10_padj. Genes significantly up- and downregulated with |FC| > 1.5 and padj < 0.1 (two-tailed Student’s *t*-test) by OVX are colored in red and blue, respectively; genes not differentially expressed are displayed in grey. *Cyp3a16*, Cytochrome P450 3A16; *Cyp4a12a*, Cytochrome P450 4A12A; *Cyp4a12b*, Cytochrome P450 4A12B. Source data are provided in^56^; processed data are provided as a Source Data file.

As shown in Fig. 1a, ERα^f/f^ mice were ovariectomized (OVX) or sham-operated (SHAM) at two months of age; starting from month 3, mice were fed for 12 weeks with a control diet (CRTL) or the iso-caloric/iso-proteic diet (*AA) modified in the composition of EAA, especially BCAA (see also Supplementary Fig. 1 and Supplementary Table 1). At the end of the dietary treatment, mice were euthanized in the early afternoon after 6 h of fasting. SHAM were euthanized in the estrous phase of the cycle, when the circulating estrogens are very low, with the aim to avoid the potentially confounding effects of the high circulating estrogens and observe the hepatic transcriptional status of cycling animals in a phase where estrogens are not dominating.

Genome-wide transcriptomic analysis, carried out by means of RNA-Seq, showed that after OVX major metabolic changes occurred in the liver (Fig. 1b, c) with a significant increase of the mRNAs encoding proteins for lipid biosynthetic processes (Supplementary Fig. 2a), acyl-CoA metabolism (Supplementary Fig. 2b), fatty acid β-oxidation (Supplementary Fig. 2c), mitochondrial synthesis of ATP (Supplementary Fig. 2d), and a decrease in mRNAs of the Wnt signaling pathway (Supplementary Fig. 2e). These changes were in line with previous reports by ours and other laboratories and pointed to a shift towards lipid anabolism^1,12,25,26^. The decrease in mRNAs encoding members of the P450 family of proteins (Fig. 1c) suggested that OVX was also associated with a decreased hepatic detoxification power.

**Fig. 2:**
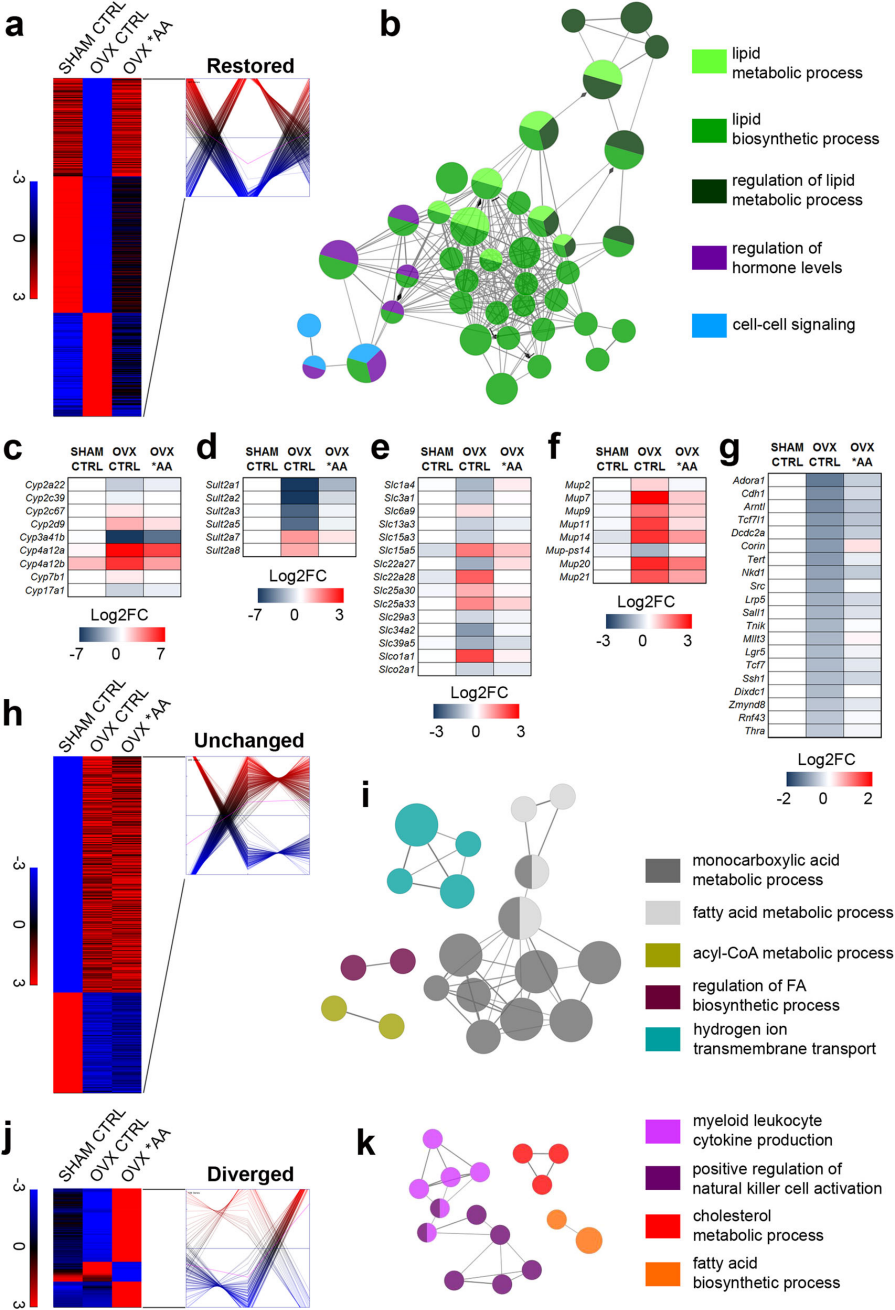
Effect of the *AA diet on liver transcriptomic profile in females lacking estrogens. By k-means clustering calculations done with Genesis, DEGs by RNA-Seq analysis (*n* = 4) related to SHAM ERα^f/f^ females fed with CTRL diet and OVX ERα^f/f^ females fed with CTRL or *AA diet were grouped in three classes according to the absolute variation in their expression profile (see also Supplementary Fig. 3). **a** Heatmaps and clusters reporting the expression of the genes which expression was affected by OVX and “restored” by *AA diet. **b** Cluster analysis of functional networks significantly enriched among the genes “restored” by *AA diet. **c**–**g** Heatmaps reporting as Log_2_FC the mean expression of the most enriched classes of genes which expression was affected by OVX and “restored” by *AA diet. **h**–**j** Heatmaps and clusters reporting the expression of the genes which expression was altered by OVX and “unchanged” (**h**) or “diverged” (**j**) by *AA diet. **i**–**k** Cluster analysis of functional networks significantly enriched among the genes altered by OVX and “unchanged” (**i**) or “diverged” (**k**) by *AA diet. Source data are provided in^56^; processed data are provided as a Source Data file.

Considering a fold change |FC| > 1.5 and an adjusted *p* value padj < 0.1, we compared the liver transcriptome of SHAM and OVX mice: in mice fed with CTRL diet the number of genes differentially expressed (DEGs) was 912, but in mice fed with *AA diet the number of DEGs was reduced of almost 2/3 (308) (Fig. 1d). The percentage of the genes that were up- or downregulated did not change significantly between the two diets (50% were the genes up-regulated with CTRL and 60% with *AA) (Fig. 1e). When we analyzed the data by Volcano plotting, we could verify that for several of the genes that remained differentially expressed with the *AA diet the extent of difference (Log_2_FC) was significantly narrowed (e.g. *Cyp3a16*, Cytochrome P450 3A16; *Cyp4a12a*, Cytochrome P450 4A12A) (Fig. 1f, g). This led us to carry out a further analysis of the diet effects in OVX with the application of the Genesis software that allowed clustering the DEGs in three groups: genes which expression was impaired by OVX and brought back to SHAM levels by the *AA diet (“restored”); genes modulated by OVX, but unmodified by the *AA diet (“unchanged”); genes, which expression was unaffected or changed by OVX and further altered by the *AA diet (“diverged”) (Fig. 2 and Supplementary Fig. 3).

In the “restored” cluster (composed of 461 of the 1,054 DEGs, 44%) (Fig. 2a–g), the most significant classes were represented by genes encoding proteins involved in the regulation of lipid metabolic processes, lipid biosynthesis, and metabolism, together with regulation of hormone levels and Wnt signaling (Fig. 2a, b). In particular, we found that the *AA diet was able to completely or almost partly restore the level of expression of gene families relevant mainly for the detoxification (P450s, Fig. 2c; sulfotransferases, Fig. 2d), transport (Fig. 2e), and the control of hepatic metabolism (*Mups*, major urinary proteins; Fig. 2f); in addition to that, the *AA diet alleviated the significant inhibition of several players of the Wnt signaling caused by OVX (Fig. 2g).

The genes “unchanged” (459/1054) by the *AA diet were related to KEGG pathways identifying oxidative phosphorylation, acyl-CoA, and fatty acid metabolic processes (Fig. 2h, i and Supplementary Fig. 4a).

The “diverged” cluster (Fig. 2j, k) was the smallest (134/1,054) and included genes encoding proteins with variegated functions, therefore difficult to be categorized in functional clusters. However, among them, we could detect 12 genes involved in the regulation of cholesterol and fatty acid metabolism (Supplementary Fig. 4b) and 17 involved in immune functions (Supplementary Fig. 4c). The expression of all of these genes appeared to over-compensate the effect of OVX.

Overall these results led us to postulate a beneficial role of the *AA diet, as it appeared to oppose the metabolic derangements consequent to OVX and, perhaps, alleviate active damaging processes occurring in the liver of OVX mice, conceivably consequence of a decreased detoxification and increased oxidative processes.

### The *AA diet mitigates body weight increase and hepatic lipid deposition consequent to ovariectomy

To further verify the extent to which the changes in gene expression observed in OVX were associated with an overall effect of the *AA diet on mice metabolism, we measured body weight (BW) and lipid content in the liver.

**Fig. 3:**
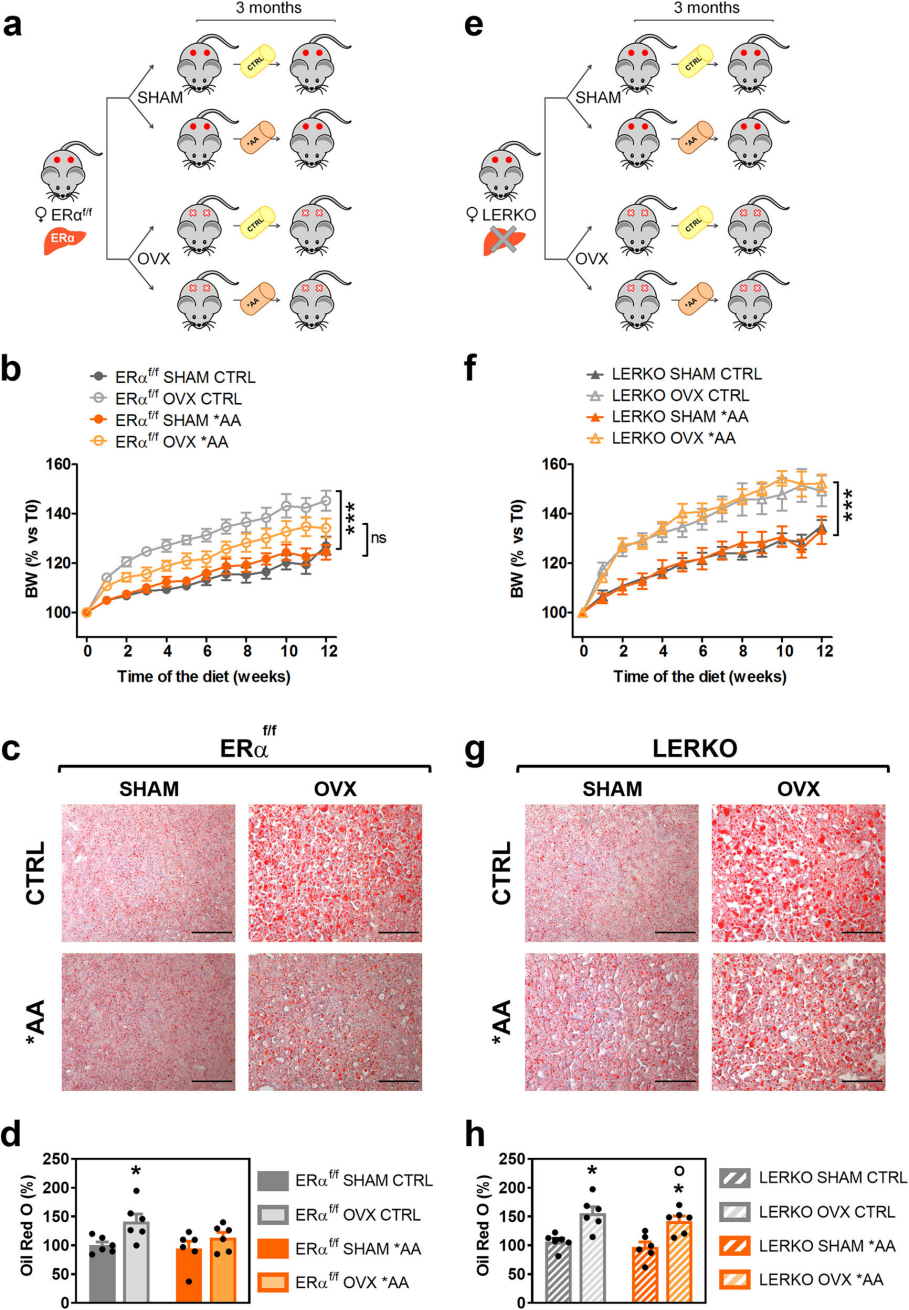
Hepatic ERα mediates the *AA-induced metabolic benefits in females lacking estrogens. **a** Experimental design adopted to evaluate the effects of *AA diet in ERα^f/f^ OVX females. **b** Body weight (BW) of SHAM and OVX ERα^f/f^ females fed with CTRL or *AA diet measured weekly for 12 weeks and expressed as percentage *versus* time 0. Data are mean values ± SEM (*n* = 8). ***p < 0.001 ERα^f/f^ OVX CTRL *versus* ERα^f/f^ SHAM CTRL by two-way ANOVA followed by Bonferroni’s *post hoc* test. **c** Representative images of the lipid deposits by Oil Red O staining in the liver tissues of SHAM and OVX ERα^f/f^ females fed with CTRL or *AA diet for 12 weeks. Orange-red: neutral fats; scale bar = 100 μm. **d** Quantification of Oil Red O staining shown in (**c**). Data are expressed as percentages of the total section areas. Data are the mean ± SEM (*n* = 6). *p < 0.05 ERα^f/f^ OVX CTRL *versus* ERα^f/f^ SHAM CTRL by two-way ANOVA followed by Bonferroni’s *post hoc* test. **e** Experimental design adopted to evaluate the effects of *AA diet in LERKO OVX females. **f** Body weight (BW) of SHAM and OVX LERKO females fed with CTRL or *AA diet measured weekly for 12 weeks and expressed as percentage *versus* time 0. Data are the mean ± SEM (n = 8). ***p < 0.001 LERKO OVX CTRL *versus* LERKO SHAM CTRL and LERKO OVX *AA *versus* LERKO SHAM *AA by two-way ANOVA followed by Bonferroni’s *post hoc* test. **g** Representative images of the lipid deposits by Oil Red O staining in the liver tissues of SHAM and OVX LERKO females fed with CTRL or *AA diet for 12 weeks. Orange-red: neutral fats; scale bar = 100 μm. **h** Quantification of Oil Red O staining shown in (**g**). Data are expressed as percentages of the total section areas. Data are the mean ± SEM (*n* = 6). *p < 0.05 LERKO OVX CTRL *versus* LERKO SHAM CTRL and LERKO OVX *AA *vs* LERKO SHAM *AA; °*p* < 0.05 LERKO OVX *AA *versus* ERα^f/f^ OVX *AA by two-way ANOVA followed by Bonferroni’s *post hoc* test. Source and processed data are provided in^56^ and as a Source Data file.

BW was measured weekly in the course of exposure to CTRL and *AA diets; as expected, in the three months of experiment (Fig. 3a), OVX ERα^f/f^ mice fed with the CTRL diet gained significantly more weight than SHAM (+13%) (Fig. 3b and Supplementary Fig. 5a). This was not the case for the mice fed with *AA diet, for which, at the end of the treatment, the weight gain was not significantly different from SHAM (Fig. 3b and Supplementary Fig. 5a). Given the fact that mice fed with CTRL and *AA diet ate comparable amount of food (Supplementary Fig. 5b and 5c), we ruled out the hypothesis that the reduced BW of OVX ERα^f/f^ mice fed with the *AA diet could be due to a reduced food intake. Conversely, the changes in feeding efficiency (FE), which increase induced by OVX was slightly mitigated by the *AA diet (Supplementary Fig. 5d), are in agreement with the concept of a different regulation of metabolism in OVX females fed with *AA diet with respect to those fed with CTRL diet.

Next, we evaluated the presence of lipid deposits in the liver. Oil red O staining indicated that a significant increase (+41%) of lipid droplets in the hepatic parenchyma was present in OVX mice fed with the CTRL diet, but not *AA diet (+9%) (Fig. 3c, d).

These results indicated that the diet enriched in EAA, especially in BCAA, was able to largely overcome the hepatic lipid metabolic imbalance induced by lack of estrogenic signaling and prevented lipid deposits as suggested by the transcriptomic data.

Very little is known with respect to the ability of AA to regulate liver metabolism; however, it is recognized that AA are essential for the full activation of the hepatic ERα^9^. This led us to hypothesize that in OVX mice *AA could compensate for the lack of estrogens by reinstating most of the hepatic ERα transcriptional activity indispensable for the lipid homeostasis characteristic of cycling females.

To verify the extent to which the hepatic ERα was involved in the response to the *AA diet, we first examined whether the diet had affected *Esr1* expression or induced the expression of genes encoding for ERβ (Estrogen Receptor β) or GPER30 (G protein-coupled receptor) (Supplementary Fig. 6). As indicated in Supplementary Fig. 6a and 6b, the diet did not affect the expression of none of these receptors. Consistent with the gene expression data, OVX and the *AA diet did not affect ERα protein content in the liver of ERα^f/f^ female mice (Supplementary Fig. 6c).

Next, we carried out additional experiments in LERKO mice:^9^ LERKO females were subjected to OVX or SHAM surgery and then fed with CTRL or *AA diet (Fig. 3e). Similar to ERα^f/f^ mice, OVX induced a significant increase in the BW of LERKO that, at the end of the dietary treatment, weighed 13% more than their SHAM counterparts. However, in LERKO OVX mice the *AA diet completely failed to oppose such a gain in BW and FE (Fig. 3f and Supplementary Fig. 5a, d). In line with these findings, liver staining with Oil red O showed that the *AA diet did not affect the significant increase in fat deposition consequent to OVX (Fig. 3g, h).

To evaluate whether OVX had systemic consequences that the *AA diet had to overcome, we measured the circulating levels of selected and relevant metabolic parameters and inflammatory markers in all the above experimental groups. As shown in Supplementary Fig. 7a, b, the levels of glucose and triglycerides (TG) were not affected by OVX and did not significantly change among the different experimental groups. By converse, the circulating levels of cholesterol (CH) were increased by OVX and normalized after *AA in ERα^f/f^, but not in LERKO mice (Supplementary Fig. 7c). This further suggested that the *AA diet was able to counteract the disruptive effects of OVX in the hepatic lipid metabolism and this effect required the presence of the hepatic ERα. Supplementary Fig. 7d shows that OVX was associated with an increase in leptin levels in both ERα^f/f^ and LERKO, but the *AA diet did not have any influence on the secretion of this hormone. Even if the leptin increase was lower in LERKO animals, we concluded that leptin has not significant central effects as food intake was almost the same in all experimental groups.

The circulating levels of interleukin 6 (IL-6), interleukin 1β (IL-β), and tumor necrosis factor α (TNFα) did not significantly change with OVX (Supplementary Fig. 7e–g), in agreement with the RNA-Seq data. This is not surprising as in mice the circulating levels of inflammatory markers are generally low and increase as a consequence of strong stimulation as, for instance, the injection of lipopolysaccharide (LPS) that we used as a control.

Altogether these findings indicated that a viable hepatic ERα is essential to enable *AA to partially but effectively re-establish the liver metabolism characterizing the cycling females in OVX females.

### The role of hepatic ERα in the response to the *AA diet

**Fig. 4:**
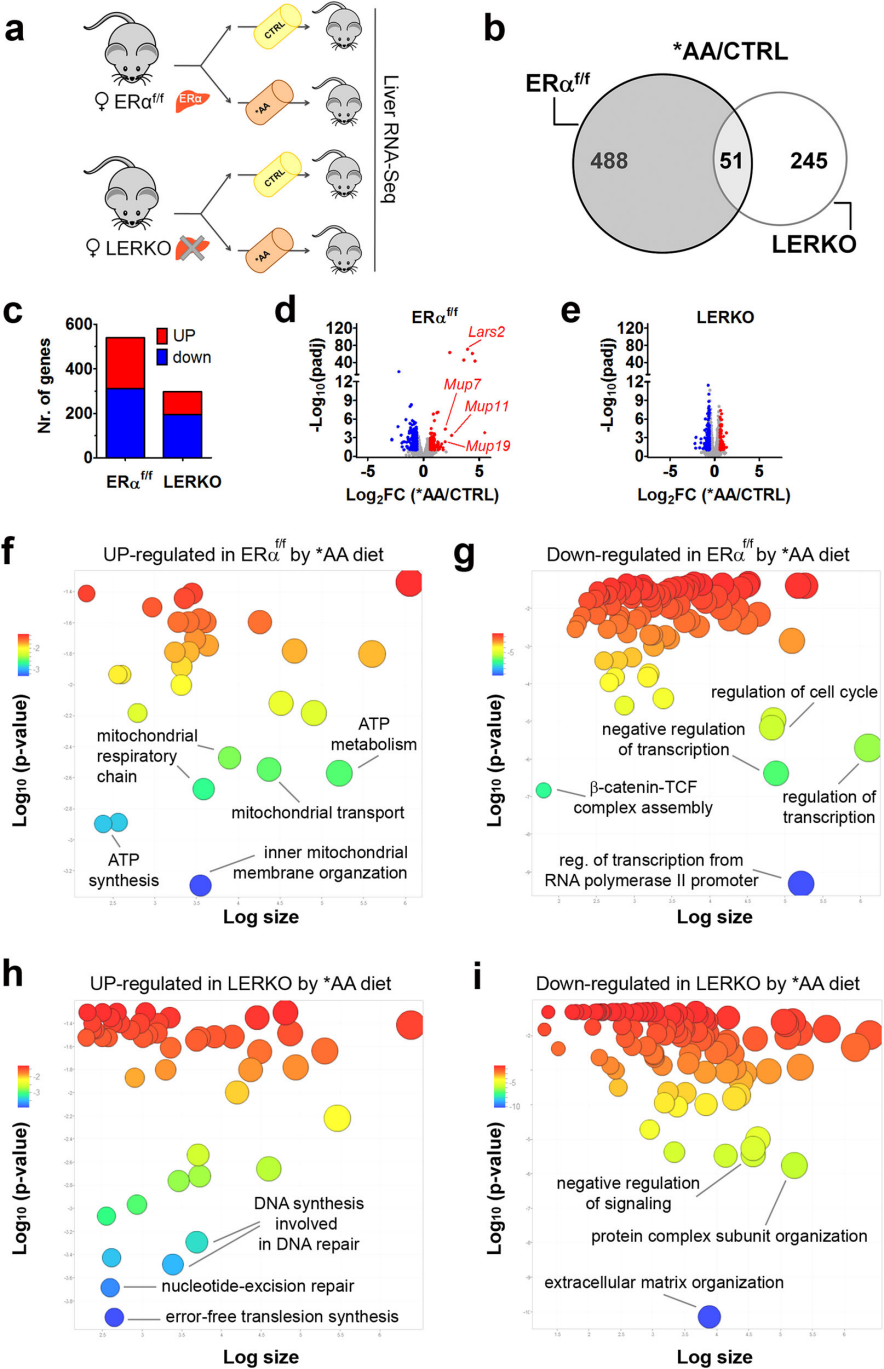
Liver ERα is necessary to maximize the hepatic metabolic response to *AA diet. **a** Experimental scheme aimed at defining hepatic ERα involvement in the response to the *AA diet. **b** Venn diagram to compare the response to the *AA diet of ERα^f/f^ (grey circle, on the left) and LERKO females (white circle, on the right). DEGs from RNA-Seq analysis (*n* = 4) were considered as significant when |FC|>1.5 and padj<0.1 (two-tailed Student’s *t*-test). **c** Distribution of genes up- and downregulated by *AA diet in the liver of ERα^f/f^ and LERKO females from RNA-Seq analysis (*n* = 4). **d**–**e** Volcano plot of DEGs identified by RNA-Seq (*n* = 4) in the liver of ERα^f/f^ (**d**) and LERKO (**e**) females fed with CTRL or *AA diet. *AA/CTRL *ratio* of gene expression is shown on the X axis as Log_2_FC and significance is displayed on the Y axis as −Log_10_padj. Genes significantly up- and down-regulated with |FC|>1.5; padj<0.1 (two-tailed Student’s *t*-test) by *AA diet are in red and blue, respectively; genes without bias are displayed in grey. *Lars2*, leucyl-tRNA synthetase 2; *Mup7*, major urinary protein 7; *Mup11*, major urinary protein 11; *Mup19*, major urinary protein 19. **f**–**i**. Gene ontology (GO) enrichment analysis generated with REVIGO. Scatterplots of GO terms obtained with Enrichr were related to the genes up- (**f**) and down-regulated (**g**) by *AA diet exclusively in the liver of ERα^f/f^ and to the genes up- (**h**) and down-regulated (**i**) by *AA diet exclusively in the liver of LERKO females. Colors indicate the *p*-value of enrichment according to the legend (in upper left-hand corner); the size of each bubble reflects the count of each term among the enriched term list. Source data are provided in^56^; processed data are provided as a Source Data file.

To better understand the metabolic role played by hepatic ERα in the response to the *AA diet, we pursued our study by investigating the differences in the liver transcriptome of ERα^f/f^ and LERKO after administration of the *AA diet (Fig. 4a). By considering a |FC| > 1.5 and a padj < 0.1, in ERα^f/f^ mice the *AA diet induced the change of the hepatic expression of 539 genes (311 upregulated and 228 downregulated) (Fig. 4b, c); LERKO response to the *AA diet was weaker and involved 296 genes, of which 195 resulted upregulated and 101 downregulated (Fig. 4b, c). The response to the *AA diet was also qualitatively different in LERKO, as only 10% of genes modulated were found to be in common with ERα^f/f^ (Fig. 4b). Furthermore, Volcano plots showed that in ERα^f/f^ the changes induced by the *AA diet were of a very large extent, particularly in the case of the genes upregulated; in fact, after the *AA diet the expression of numerous genes changed of several orders of magnitude with a very high level of significance (Fig. 4d). The genes most affected were *Lars2* (leucyl-tRNA synthetase 2), encoding an enzyme involved in the aminoacylation of tRNA, and several major urinary proteins (i.e. *Mup7*, *Mup11*, *Mup19*) involved in the regulation of glucose and lipid metabolism^27^. Very dissimilar was the effect of the *AA diet in LERKO, for which the extent of the response was much more restricted, not only considering the number of genes involved, but also in terms of significance (−Log_10_padj) and intensity of the effect (Log_2_FC) (Fig. 4e).

In SHAM ERα^f/f^ females (Fig. 4f, g), *AA diet mostly affected the expression of genes associated with ATP synthesis/metabolism and mitochondrial respiratory chain (Supplementary Fig. 8a), β-catenin TCF assembly pathway (Supplementary Fig. 8b), cell cycle (Supplementary Fig. 8c), and regulation of transcription (Supplementary Fig. 8d). Differently from ERα^f/f^, the effect of the *AA diet in LERKO did not include genes relevant for increased mitochondrial respiration and for augmented transcription but appeared to be circumscribed to DNA repair (Fig. 4h) and regulation of extracellular structural proteins (Fig. 4i). The analysis of the identity of the DEGs revealed that in the LERKO fed with the *AA diet the pathways regulated were compatible with an AA-dependent activation of the TORC1 complex, showing an increase of DNA repair mechanisms (Supplementary Fig. 8e) and a decrease of TGFβ signaling (Supplementary Fig. 8f)^28^ and ability to induce the genes for collagen deposition (Supplementary Fig. 8g) and organization of the extracellular matrix (Supplementary Fig. 8h). These results suggest the likelihood that, in the absence of ERα, liver response to *AA diet should be compatible with the expected activation of the TORC1 complex, but when the receptor is present the effect of this diet is more compatible with an activation of the ERα signaling, with consequent reflection on metabolic pathways where the receptor is known to be very active.

**Fig. 5:**
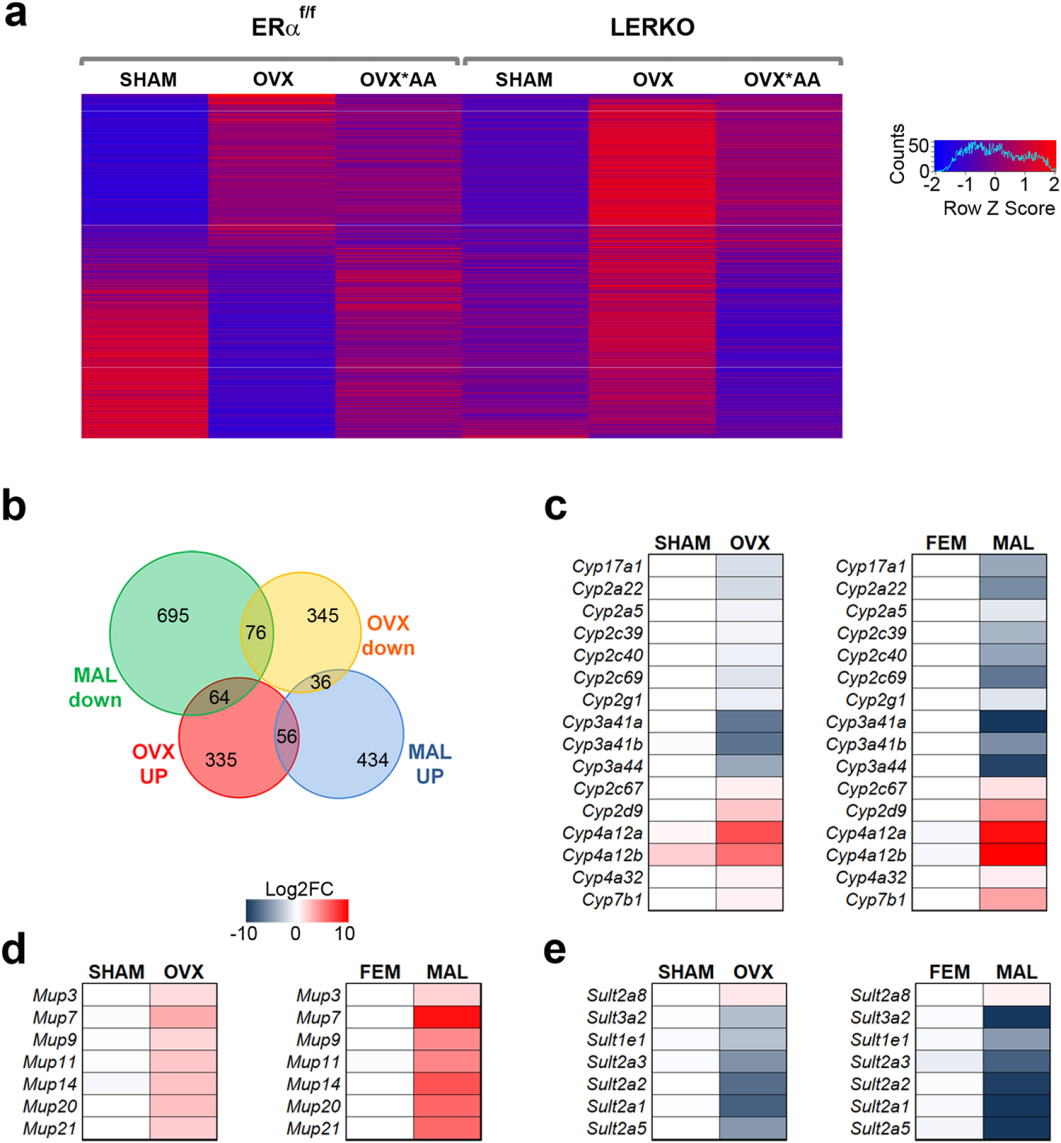
Comparison of hepatic transcriptomes from different female genotypes and from males. **a** Heatmap showing overall the DEGs from RNA-Seq analysis (*n* = 4) related to OVX and/or diet in ERα^f/f^ and LERKO females. **b** Venn diagram summarizing the overlap between DEGs identified by RNA-Seq (*n* = 4) in the liver of male, fertile female and OVX female ERα^f/f^ mice. **c**–**e** Heatmaps reporting as Log_2_FC the mean expression (*n* = 4) of the most enriched class of genes differentially expressed in the livers of OVX ERα^f/f^ females and in the livers of ERα^f/f^ males with respect to fertile ERα^f/f^ females. Source data are provided in^56^; processed data are provided as a Source Data file.

These findings were further supported by a comparative analysis of all of the 1,305 genes affected by OVX with/without *AA in ERα^f/f^ and LERKO that is recapitulated by the heatmap in Fig. 5a (obtained with a mean of four animals/group). By comparing the extent of the expression of these genes in ERα^f/f^ and LERKO, we could conclude that the ablation of the hepatic ERα per se did not have dramatic consequences (as it involved only 107 DEGs). OVX in LERKO affected only 174 genes that were mostly upregulated, therefore it did not induce the major changes observed in the ERα^f/f^, for which the color code clearly showed an inversion of the rate of expression of most of the genes analyzed (912/1,305). Most relevant, however, was the fact that, contrary to what observed in the OVX ERα^f/f^, in the OVX LERKO the *AA diet failed to realign the pattern of gene expression to that of ERα^f/f^ mice (Fig. 5a and Supplementary Fig. 9). More interestingly, in the OVX LERKO the *AA diet induced a transcriptomic profile almost superimposable (97%) to that of the OVX ERα^f/f^ when fed with CTRL diet, being the DEGs between OVX ERα^f/f^ CTRL-fed and OVX LERKO *AA-fed only 40 (Fig. 5a). These latter results could be expected as the *AA diet was unable to tackle the metabolic disturbances induced by OVX in terms of body weight gain and liver lipid deposition.

Thus, in the absence of ovarian functions, the AA present in the diet had to activate the unliganded hepatic ERα, unless the *AA diet could augment the steroidogenic power of organs other than the ovaries (such as the brain of the adipose tissue). To rule out this hypothesis, we measured the uterus weight that is a well-known and very sensitive marker of circulating estrogenic compounds. As shown in Supplementary Fig. 10, OVX decreased dramatically the uterus weight (that is very reproducible in SHAM because we used females at the same stage of the reproductive cycle), but, very clearly, the *AA diet did not affect ERα^f/f^ and LERKO uterus weight.

All together these analyses provided further indication of the relevance of the hepatic ERα as a sensor and a modulator of hepatic metabolism.

### Defeminization of liver metabolic profile after ovariectomy

We next posed a different question and asked the extent to which circulating estrogens play a role in maintaining the sexual dimorphism of the hepatic transcriptome^2,3^. In fact, it is well known that the liver is a sexually dimorphic organ and it has been proposed that the lower incidence of metabolic and cardiovascular diseases that characterizes fertile females is associated with the overall tight control that the female liver exerts over energy metabolism^2,3^. The fact that, after the cessation of ovarian functions, women rapidly lose their metabolic advantage over males^1^ suggests that, in case of ovarian failure, female and male liver metabolism become comparable.

When we compared the genes differentially regulated in the liver after OVX with those differently expressed in males and females, we found that about 25% of the over 900 genes responsive to OVX were sexually dimorphic^8^ (Fig. 5b). Interestingly, among the DEGs shared by/between OVX females and males, most of them had very close expression, supporting the view that circulating estrogens have a major role in preserving hepatic sexual dimorphism.

Most surprisingly, the large majority of the genes expressed similarly to males after OVX were concentrated in three families: *Cyp450, Mups,* and *Sult* (Fig. 5c–e). *Cyp* and *Sult* are gene families mostly involved in the detoxification and catabolism of steroids while *Mups* preside over energy metabolism.

This indicated that, together with a greater control of lipid metabolism, the liver of fertile females has a greater detoxification ability compared to that of males and OVX females: this might explain the different susceptibility of males and females to hepatic damages and cardiovascular consequences^3^ and why females, after the cessation of ovarian functions, become more susceptible to hepatic and cardiovascular disorders^7,14,21^.

## Discussion

**Fig. 6:**
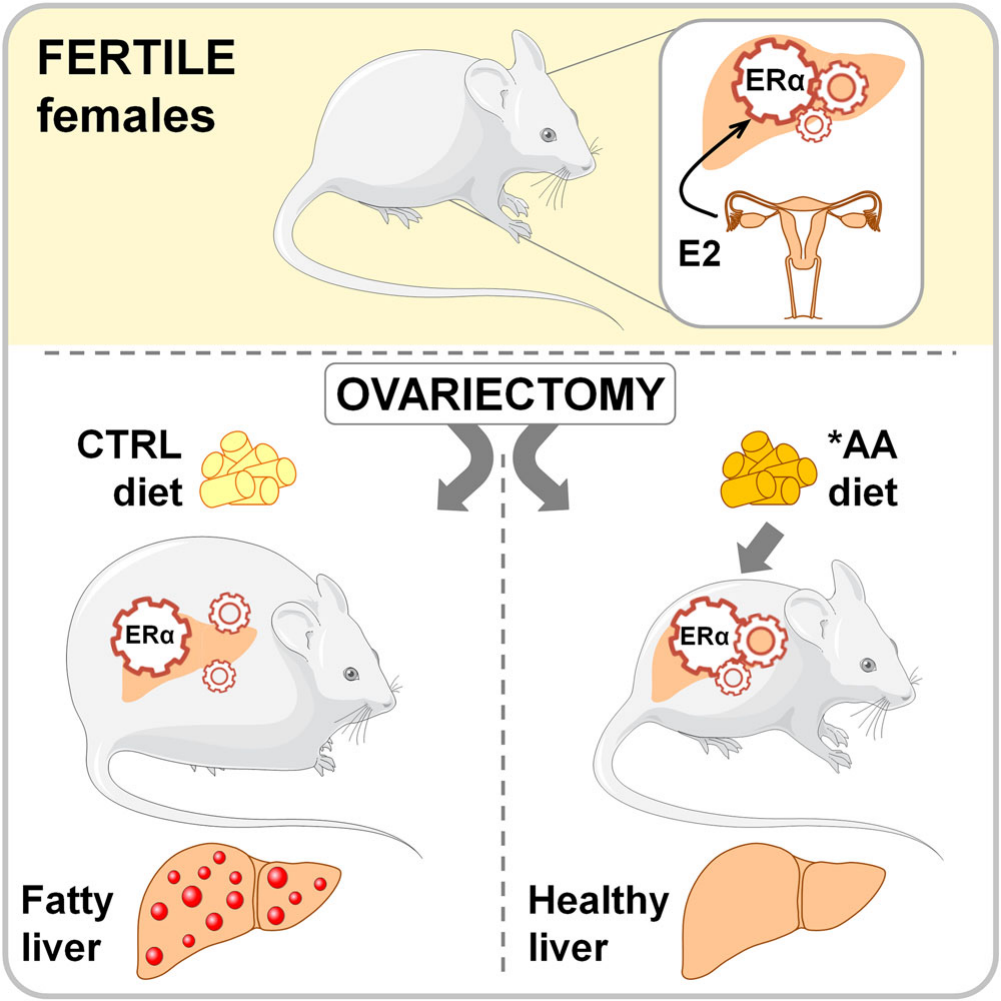
The AA* diet induces metabolic benefits in OVX females that are mediated by the hepatic ERα. In female mice, ovariectomy induces liver lipid deposition and body weight increase. The *AA diet reinstates a more balanced hepatic metabolism contributing to avoid fat deposition in the liver and to reestablish the lean phenotype typical of the intact females. This phenomenon is not observed in LERKO mice, suggesting a pivotal role of the hepatic ERα in the control of lipid and energy homeostasis in females. Image was modified from Servier Medical Art, licensed under a Creative Common Attribution 3.0 Generic License. http://smart.servier.com/.

The main findings of this study are twofold: a diet enriched in EAA, and BCAA in particular, opposes the dysmetabolism associated with the loss of ovarian functions to the point to limit the OVX-induced body weight gain and deposition of lipids in the liver; this effect is mediated by the hepatic ERα (Fig. 6). This latter result points to the major role played by the liver and by the hepatic ERα in the maintenance of energy homeostasis in females.

In the course of evolution, well-harmonized and intertwined programs between liver and reproductive organs had to be perfected to secure that the reproductive functions were successfully executed and maintained under the strict control of nutritional cues^9,12,13^. In female mammals, this was achieved *via* the hepatic ERα that, upon ovarian and nutritional stimulation, finely tunes liver metabolic programs in relation to the reproductive needs and nutrients availability^9,13^. Accordingly, it is not surprising that, in the absence of circulating estrogens, the declining ERα activity has significant metabolic repercussions^1,7,12,14,15^.

By the analysis of the hepatic transcriptome, the present study gives a global overview of the liver response to the lack of ovarian stimulation. As expected, after OVX liver lipid metabolism and mitochondrial activity change significantly leading to the accumulation of fats in the liver; in addition, Wnt signaling appears to be involved in the estrogen-dependent control of liver metabolic functions (Figs. 2g and 4g), in line with what previously suggested by other authors^29^.

The observation that the *AA diet was able to limit the increase in body weight that is associated with OVX was not anticipated. In fact, it is well known that the ERs are present and functionally active in all metabolic organs, including the brain, thus the increased body weight after OVX could be associated with a summation of events occurring in all these organs were the ERs cease to be stimulated. Hence, the *AA diet could have affected the metabolism of all these organs. The present study argues against this view, by demonstrating that the ablation of liver ERα alone was sufficient to abolish the *AA-dependent inhibition of OVX-induced increase of body weight. This provides strong evidence that the hepatic ERα, through the control of liver lipid metabolism, has a key role in the systemic response to OVX. It is important to underline that the simple ablation of the hepatic ERα did not induce any measurable change in BW, thus BW per se could not explain any of the results obtained: this was also demonstrated with a series of correlation analyses (not shown). All of the above observations, together with prior studies^8,9,17^, indicate the necessity of a functional liver ERα for the metabolic flexibility that females have in response to dietary changes.

It remains to be elucidated how the *AA diet could regulate liver activities when the hepatic ERα is expressed. As shown in Supplementary Fig. 1c, the increased content of selected EAA in the diet was mostly at the expenses of not-essential AA that could be synthesized by the mice. The AA present in the diet could affect liver activities directly, or *via* the metabolites generated in other metabolic organs, mainly the skeletal muscle^30^. We know that, at cellular level, the nutrient-sensing pathway is the mechanistic target of rapamycin, mTOR^31,32^. mTOR is a serine/threonine-protein kinase organized in a protein regulator complex, TORC1, that promotes anabolic and represses catabolic programs^33^. In the absence of liver ERα (LERKO mice), the *AA diet regulates the hepatic expression of about 300 genes, which function is quite compatible with the activation of the TORC1 pathway because involved in DNA repair^34^ and alteration of matrix deposition. When ERα is present, the effects of the *AA diet implicate a much larger number of genes (more than 500) relevant for functions completely different than in LERKO. Notably, these latter functions (increased mitochondrial respiration, augmented transcription, modulation of apoptosis, and inflammation) are very much in line with ERα activation. What these data are clearly indicating is that, in the presence of a viable liver ERα, the activity of the sensor(s) of AA is tremendously amplified and involves several other pathways. To explain these results, we can speculate that the unliganded ERα is activated by a mTOR-dependent phosphorylation as indicated by our previous publications and recently demonstrated by Marina Holz^35,36^. We cannot rule out other post-translational modifications that may enable the receptor to regulate the activity of a wider range of signaling molecules^16^.

In addition, the mRNAs involved in Wnt signaling were decreased significantly in OVX and rescued by the *AA diet in ERα^f/f^ mice only, suggesting an interaction between Wnt and ER signaling pathways, which could occur possibly through a modulation of FOXO (forkhead box O) activity or by the increased ATP production^37^ and a potential involvement of Wnt in the control of hepatic metabolism^38^. The involvement of mTOR and Wnt signaling programs in ER-mediated metabolic response to the *AA diet suggests that, in the mammalian liver, the ancestral mechanisms responsible for longevity and reproduction are maintained^1^. If this was the case, the findings of this study might likely be translated to women pointing to a nutrition-based mean to attenuate pathologies associated with women ageing. In fact, it is well known that with menopause the lack of the regulatory action of estrogens makes women more susceptible to metabolic derangements and to an increased risk of developing metabolic diseases, such as metabolic syndrome (MetS), NAFLD, and diabetes^1,7,14,20^. Such a risk is particularly elevated in overweight and obese post-menopausal women that have lost the protective effects of estrogens^1,3,21,39^. In this scenario, therapies aimed at counteracting the negative effects associated with menopause should be considered the main goal to reach for women health during ageing. In the absence of precise insights on the mechanisms responsible for the initial metabolic alterations occurring after the cessation of ovarian functions, current hormone replacement therapies were simply meant to re-establish higher plasma levels of estrogens^40,41^, without taking into account the necessity of their oscillation for a perfect restoration of the hormone physiological effects^12,13,42^.

By providing a clearer view of the significance, relevance, and mechanisms of activation of the hepatic ERα in the orchestration of female metabolism, the present study offers new bases for the design of treatment of the post-menopause that could rely heavily on dietary interventions. Interestingly, previous Authors studied the effect of AA-enriched diets in breast cancer patients and no undesired effects, including an increased risk of cancer recurrence, were shown^43–45^. It is important to underline that, in our prior experimentation^9^, AA administration appeared to activate the liver ER in a prevalent manner; this would be particularly relevant, as it is well known that estrogenic compounds in post-menopausal women may be associated with and increased risk of breast cancer. In addition, of course, appropriate clinical experimentation should be carried out to demonstrate the maintenance of the mechanisms unraveled by our study in mice.

In conclusion, in this study the hepatic ERα stands as a valuable target for preventive therapies aimed at maintaining the hepatic functions necessary for women metabolic health: our findings suggest that, together with pharmacological therapies aimed at specifically activating the hepatic ERα, appropriate dietary interventions might prove very successful to limit the metabolic derangements consequent to ovarian failure and their countless consequences for women health in the course of ageing.

## Methods

### Animals

In the present study, we used syngenic ERα floxed (ERα^f/f^) and LERKO (liver-specific ERα knockout)^9^, both C57BL/6 J strain. Mice were fed *ad libitum* with a standard diet (4RF21 standard diet, Mucedola) and provided with filtered water. The animal room was maintained within a temperature range of 22–25 °C, relative humidity of 50 ± 10%, and under an automatic cycle of 12-h light, 12-h dark (lights on at 07:00 a.m.). At two months of age, mice were anesthetized with a s.c. injection of 70 μL of ketamine (109.2 mg/kg Ketavet 100, Intervet) and xylazine (8.4 mg/kg Rompun, Bayer) solution and then ovariectomized (OVX) or sham (SHAM) operated. After four weeks, animals were assigned to a specific experimental group and fed with a control diet (CTRL) or a diet enriched in EAA and BCAA (*AA, see also Supplementary Fig. 1 and Supplementary Table 1) for 12 weeks. The diet was selected through a literature search because demonstrated to be one of the most balanced, safe, and palatable for the mice. At the end of the experiment, mice were six months old. SHAM female mice were collected when in the estrus phase with vaginal smears done at 9:00 a.m. In all experiments, mice were euthanized in the early afternoon after six hrs of fasting to avoid potential confounding effects due to the circadian rhythm or feeding status^9^. For each experimental group, *n* = 8 mice; the experiment was repeated twice. All animal experimentation was done in accordance with the ARRIVE and European guidelines for animal care and use of experimental animals. The study was approved by the Italian Ministry of Research and University and a Departmental panel of experts was responsible for the control of all handling and surgical protocols.

### RNA-sequencing sample and library processing

RNA from liver of control (ERα^f/f^) and LERKO mice was isolated with TRIzol (Invitrogen) and purified using the RNeasy minikit protocol (Qiagen), according to the manufacturer’s instructions. RNA Quality Control was performed with the RNA ScreenTape (Agilent, Santa Clara, CA) on Agilent 4200 TapeStation System (Agilent, Santa Clara, CA). The RNA Integrity Number (RIN) was determined for every sample and all samples were considered suitable for processing if RIN > 7.5. RNA concentration was spectrophotometrically estimated using Eppendorf BioSpectrometer Fluorescence instrument. Sequencing libraries were prepared using the TruSeq® Stranded mRNA Library Prep (Illumina, San Diego, CA) with an input of 700 ng of total RNA. Final libraries were validated and quantified with the D1000 ScreenTape on 4200 TapeStation System. Pooled libraries were sequenced on Illumina HiSeq platform, producing 2 × 150 bp paired end reads.

### Transcriptomics data analysis

Raw sequencing reads were processed for quality check using FASTQC (v0.11.5) (http://www.bioinformatics.babraham.ac.uk/projects/fastqc/). Pre-alignment data processing, including trimming and adapter removal was not performed as it was not necessarily due to the high quality of data. Raw paired end reads were then mapped to the mouse reference genome (Gencode GRCm38 primary assembly M20 and the associated GTF annotation file) using STAR aligner (v2.5.2a)^46^. Raw read counts were generated using STAR using the quantMode TranscriptomeSAM option. Aligned data were manipulated using Samtools (v1.1)^47^.

In particular, we evaluated coverage across gene body, transcriptome profile efficiency (percentage of reads mapping to exons), samples correlation matrices, and number of detected genes in order to identify possible contaminations, mapping failures, and obvious outliers. We quantified gene expression by using the quantmode GeneCounts option in STAR. The counts produced coincide with those produced by htseq-count^48^ with default parameters. Samples counts were merged into a single gene counts matrix (32 samples, 46983 genes) which was used as input for differential expression analysis. The statistical analysis was performed by using DESeq2 package (v1.30.0)^49^, testing (Wald Test) group *vs* group accordingly to experimental design. Unless otherwise stated, a threshold of 0.05 was applied to False Discovery Rate (FDR) adjusted *p* values in order to select the differentially expressed genes (DEGs) to use in downstream analysis. Exploration data analysis (clustering and principal component analysis - PCA) was performed using build in functions in DESeq2 package.

Venn diagram showing the number of DEGs in mouse liver was made with Bioinformatics & Evolutionary Genomics software (http://bioinformatics.psb.ugent.be/webtools/Venn/). Cluster analysis of functional networks significantly enriched in the liver was performed by using the Cytoscape (v3.7.1) plug-in ClueGO^50^ with the following parameters: ontology: GO, biological process: all (update 04.09.2018); enrichment: right-sided hypergeometric test; GO tree level: 3–15; *p* value: <0.05; *p* value correction: Bonferroni; GO term restriction: three genes minimum, 4% genes; kappa score: 0.4; initial group size: 2; group merge: 50%; leading group term: highest significance. Gene ontology (GO) analysis on DEG lists was performed using the David (v6.8)^51^, Enrichr (http://amp.pharm.mssm.edu/Enrichr/)^52^, REVIGO (http://revigo.irb.hr/)^53^ and Genesis (v1.8.1)^54^ (see also Table 1).

**Table 1:** Transcriptomics tools and deposited data.

**Software and algorithms utilized**		
Fastqc (v0.11.5)	(http://www.bioinformatics.babraham.ac.uk/projects/fastqc/)	http://www.bioinformatics.babraham.ac.uk/projects/fastqc/
STAR (v2.5.2a)	^46^	https://github.com/alexdobin/STAR
Samtools (v1.1)	^47^	https://sourceforge.net/projects/samtools/
DESeq2 (v1.30.0)	^48^	https://bioconductor.org/packages/release/bioc/html/DESeq2.html
Bioinformatics & Evolutionary Genomics	http://bioinformatics.psb.ugent.be/webtools/Venn/	http://bioinformatics.psb.ugent.be/webtools/Venn/
Cytoscape/ClueGO (v3.7.1)	^50^	http://apps.cytoscape.org/apps/cluego
David (v6.8)	^51^	https://david.ncifcrf.gov/
Enrichr	^52^	http://amp.pharm.mssm.edu/Enrichr/
REVIGO	^53^	http://revigo.irb.hr/
Genesis (v1.8.1)	^54^	http://genome.tugraz.at
Shinyheatmap	^57^	http://shinyheatmap.com/
**Deposited Data**		
Mouse reference genome, GRCm38	Genome Reference Consortium	https://www.ncbi.nlm.nih.gov/grc/mouse
Gencode GRCm38 primary assembly M20 and the associated GTF annotation file	Gencode	https://www.gencodegenes.org/mouse/release_M20.html
Raw data	^56^	https://www.ncbi.nlm.nih.gov/bioproject/PRJNA778593
**Commercial kits**		
TruSeq® Stranded mRNA Library Prep	Illumina Italy S.r.l.	20020594
RNA ScreenTape	Agilent Technologies Italia S.p.A.	5067-5576

### Liver histology

The left lobe of the liver was fixed in 10% neutral formalin solution (Sigma-Aldrich) overnight at 4 °C, cryopreserved in a 30% (w/v) sucrose solution for 24 h at 4 °C, and stored at −80 °C. Seven μm-thick liver sections were cut with a refrigerated microtome (Leica), collected on poly-L-lysine–coated glass slides, and stored at −80 °C until staining. Oil Red O lipid stain was done with a 5% solution of Oil Red in Propylene glycol for 10 min, in a heater, at 60 °C, followed by a 5 min of wash in 85% Propylene glycol solution (Oil Red O and Propylene glycol - Sigma). Hematoxylin–eosin (H&E) counterstaining was done on frozen slides with Mayer hematoxylin (Bio-Optica) for 1 min and, after water rinsing, with 1% eosin aqueous solution (Bio-Optica) for 4 min. After staining, the slides were cleared in xylenes and cover slipped with xylenes-based mounting medium (Eukitt, Bio-Optica). The liver sections were evaluated in blind by light microscopy. Images of the stained sections were captured using Microscope Axioscop2 mot plus (Zeiss). Quantitative analysis of lipid droplet-stored triglycerides was done using ImageJ imaging software^55^.

### Quantification and statistical analysis

All statistical analyses were done using GraphPad Prism 5.0 (GraphPad Software). Multiple testing comparisons were done by two-way ANOVA followed by Bonferroni’s *post hoc* test; two-tailed Student’s *t-*test was used for comparisons between two experimental groups. All data are expressed as mean ± SEM. A *p* value less than 0.05 was considered statistically significant. All statistical parameters are in the figure legends.

### Reporting summary

Further information on research design is available in the Nature Research Reporting Summary linked to this article.

## Supplementary information

Supplementary information

Reporting Summary

## Data Availability

Mouse reference genome GRCm38 is available at the web page https://www.ncbi.nlm.nih.gov/grc/mouse. Gencode GRCm38 primary assembly M20 and the associated GTF annotation file are available at the web page https://www.gencodegenes.org/mouse/release_M20.html. The RNA-Seq raw data generated in this study have been deposited in the OSF database https://www.ncbi.nlm.nih.gov/bioproject/PRJNA778593 (BioProject PRJNA778593)^56^. The processed RNA-Seq data generated in this study and other source data are provided in the Source Data file. Source data are provided with this paper.

## Source data

Source Data
