# Supplementary material for: Dietary essential amino acids restore liver metabolism in ovariectomized mice via hepatic estrogen receptor α
Source: Nat Commun. 2021 Nov 25;12:6883. doi: 10.1038/s41467-021-27272-x (PMC8617046; doi:10.1038/s41467-021-27272-x)
Supplement: Supplementary file 1 — Supplementary information [file 41467_2021_27272_MOESM1_ESM.pdf]

SUPPLEMENTAL INFORMATION

Supplementary Fig. 1

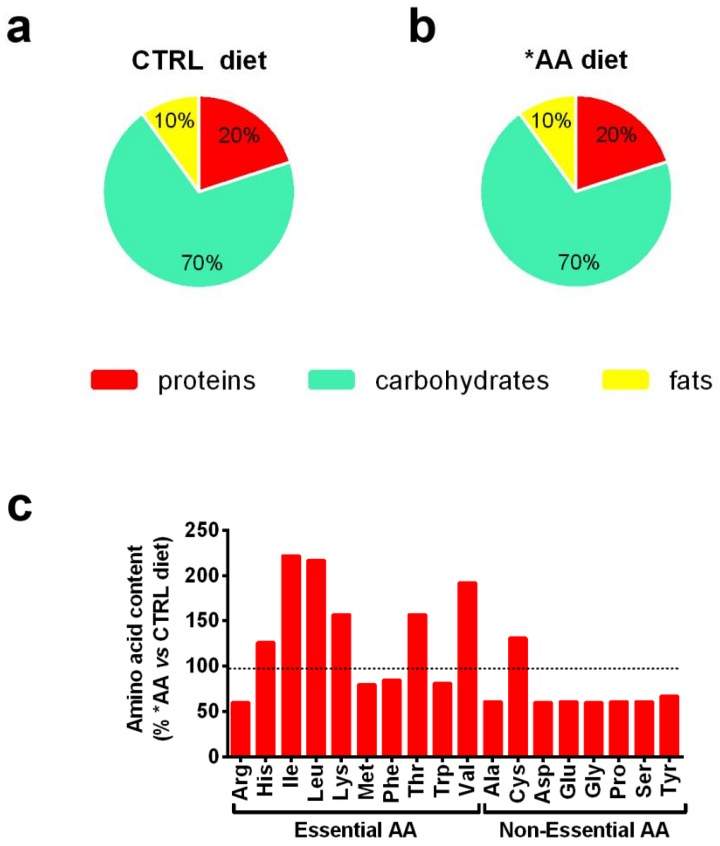

Supplementary Fig. 1. Composition of CTRL and *AA diets. (a, b) Pie charts showing the composition of CTRL (**a**) and *AA (**b**) diets as Kcal %. **c** Amino acid content of the *AA diet expressed as percentage *versus* CTRL diet. Source data are provided as a Source Data file.

Supplementary Fig. 2

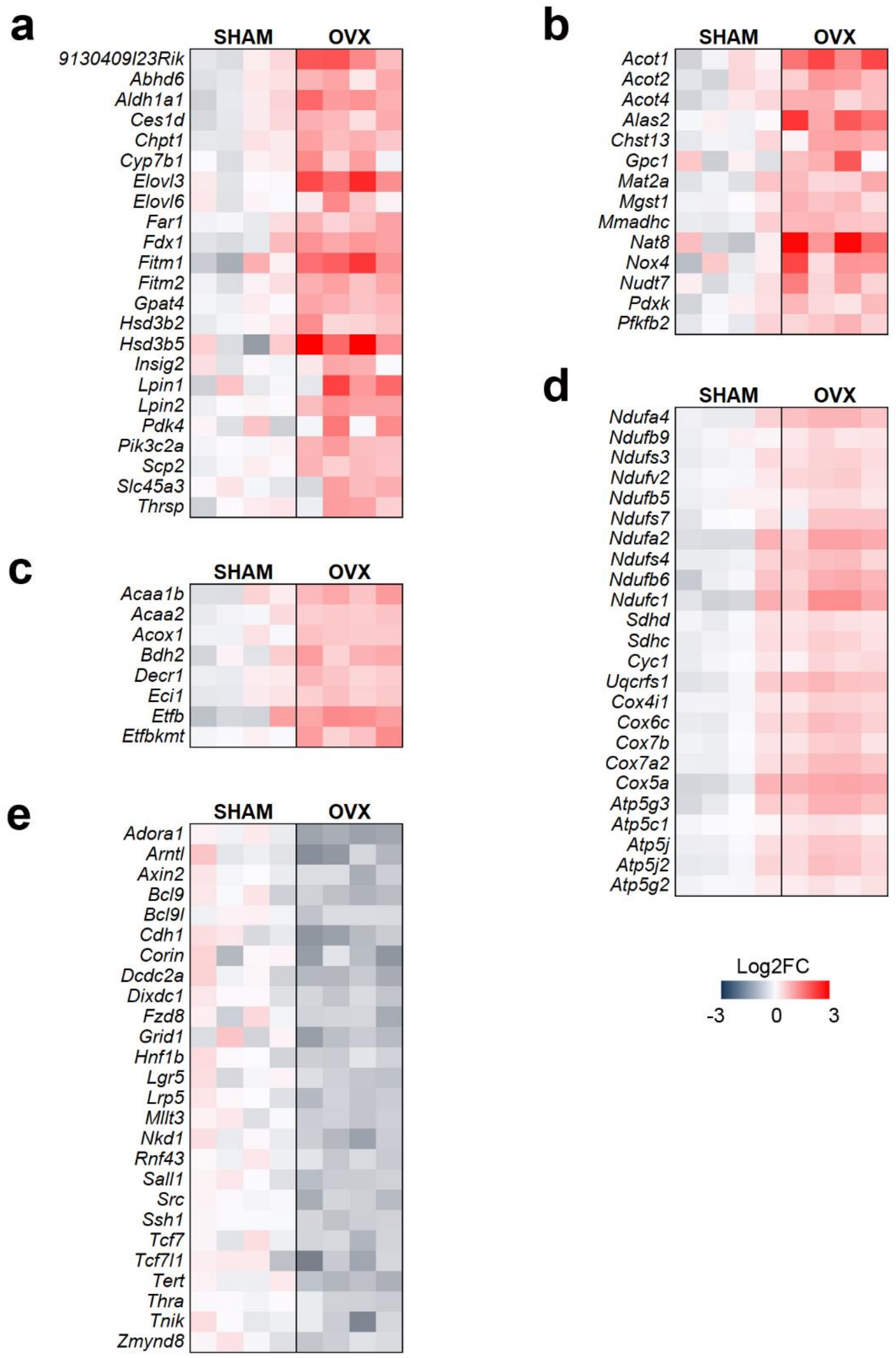

Supplementary Fig. 2. Enrichment analysis of DEGs measured in the liver of OVX ERα$^{f/f}$ females. a-e Heatmaps reporting as Log_2FC the expression of genes altered by OVX in the liver of ERα$^{f/f}$ females and associated with lipid biosynthetic process (**a**), acyl-CoA metabolic process (**b**), fatty acid β-oxidation (**c**), mitochondrial ATP synthesis coupled electron transport (**d**), and Wnt signaling pathway (**e**) from RNA-Seq analysis (n=4). Source and processed data are provided in [1] and as a Source Data file.

Supplementary Fig. 3

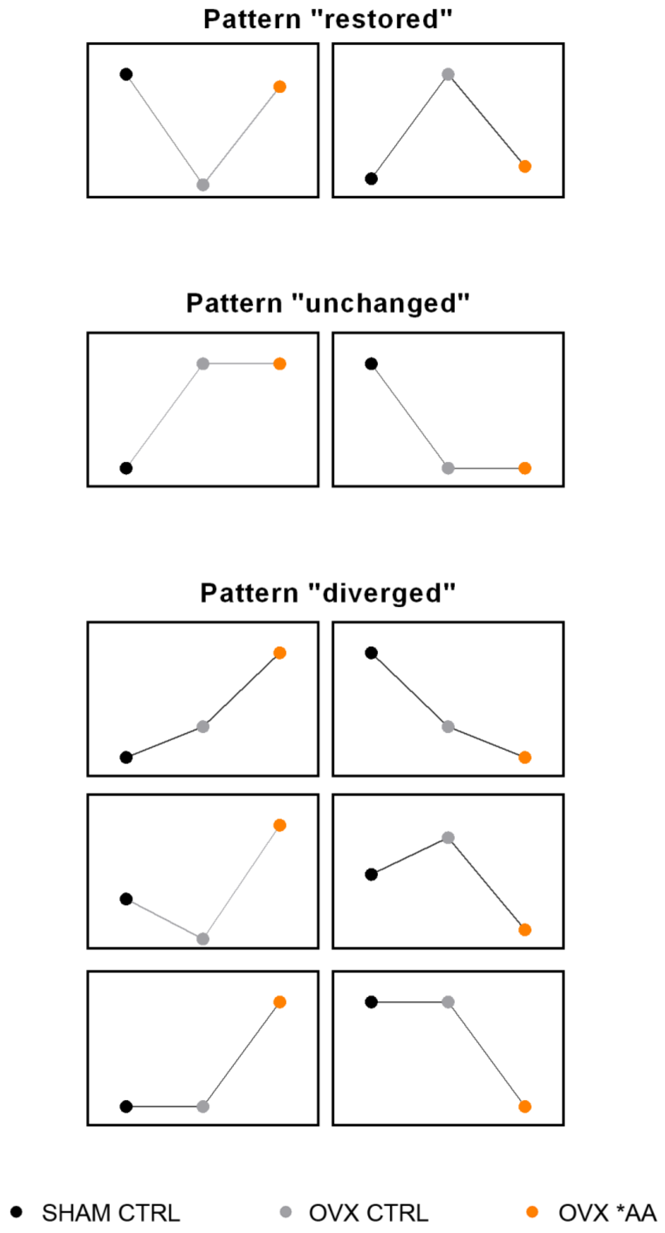

Supplementary Fig. 3. Theoretical expression patterns of genes altered by OVX and/or by *AA diet in the liver of ERα$^{f/f}$ females. Theoretical expression profile of DEGs obtained by RNA-Seq analysis performed in the liver of SHAM ERα$^{f/f}$ females fed with CTRL diet and OVX ERα$^{f/f}$ females fed with CTRL or *AA diet. Gene expression is shown in vertical axis; lines and dots represent samples and their average expression. DEGs can be grouped in 3 classes according to the absolute variation in their expression profile: to the ñrestoredò class belong genes, whose expression is impaired by OVX and re-established by *AA diet to the levels measured in the liver of SHAM females; to the ñunchangedò class belong genes, whose expression is affected by OVX and unmodified by *AA diet; to the ñdivergedò class belong genes, whose expression is unaffected or changed by OVX and further altered by *AA diet.

Supplementary Fig. 4

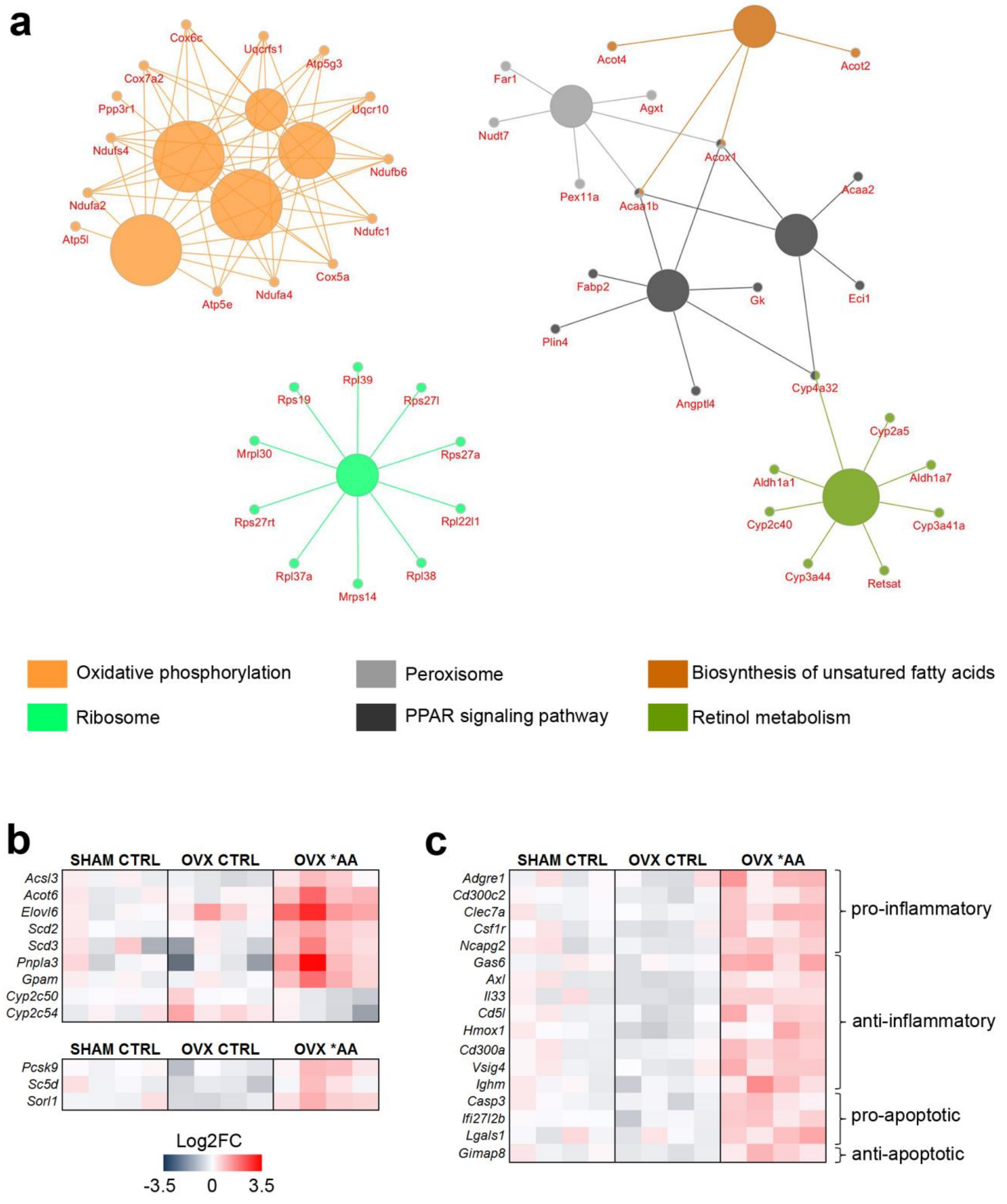

Supplementary Fig. 4. Enrichment analysis of DEGs whose expression resulted ñunchangedò or ñdivergedò by *AA diet in the liver of OVX $ER\alpha^{f/f}$ females. a Cluster analysis of KEGG networks from RNA-Seq (n=4) significantly enriched among the genes ñunchangedò by *AA diet in the liver of OVX $ER\alpha^{f/f}$ females. **(b, c)** Heatmaps reporting as Log_2FC the expression of the most enriched genes among the ñdivergedò class: fatty acid and cholesterol metabolic process (**b**) and regulation of immune response (**c**). Source and processed data are provided in [1] and as a Source Data file.

Supplementary Fig. 5

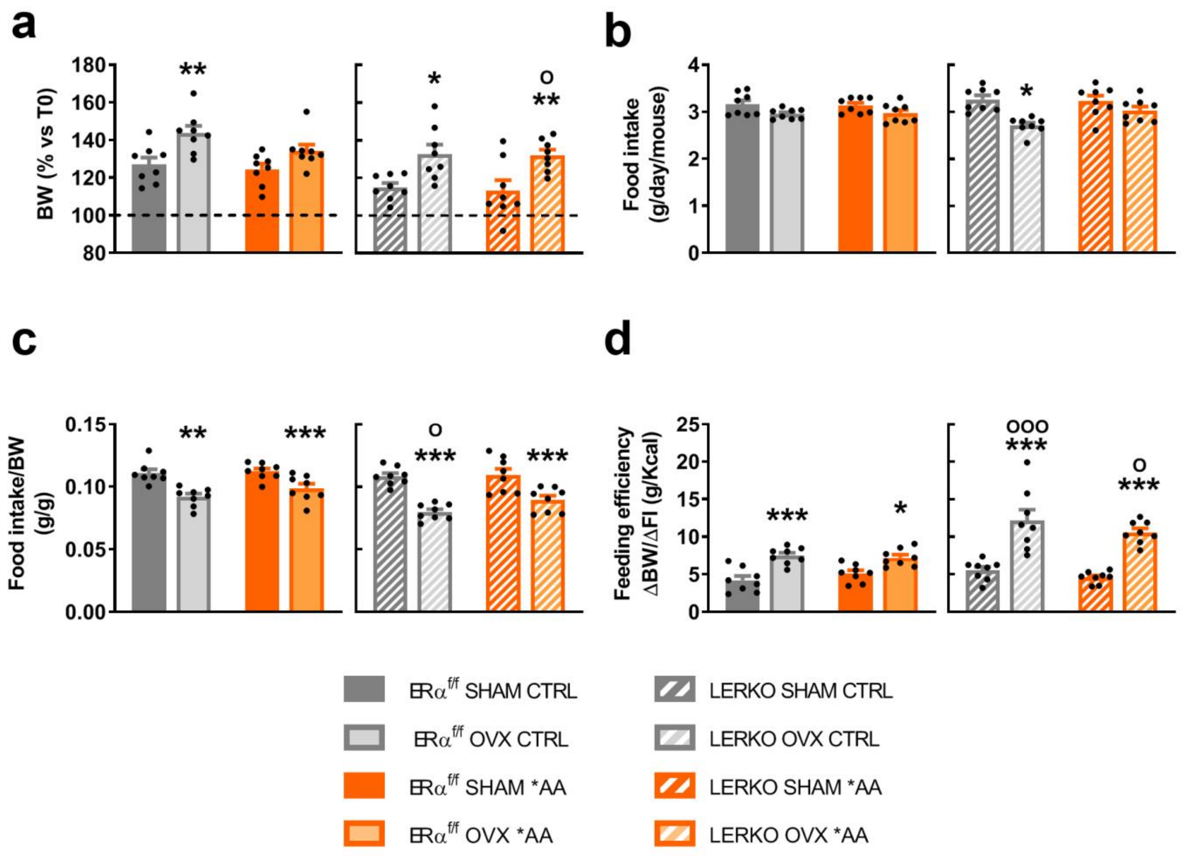

Supplementary Fig. 5. Systemic response to *AA diet. a Body weight (BW) (expressed as % *versus* time 0) of SHAM and OVX ERα$^{f/f}$ and LERKO females fed with CTRL or *AA diet measured at the end of the 12 weeks long experiment. Data are shown as mean ± SEM (n = 8). *p<0.05 and **p<0.01 OVX *vs* SHAM; °p<0.05 LERKO OVX *AA *vs* ERα$^{f/f}$ OVX *AA by two-way ANOVA followed by Bonferroniȏs *post hoc* test. **b** Food intake expressed as g/day/mouse measured along the 3 month-long experiment. Data are shown as mean ± SEM (n = 8). *p<0.05 LERKO OVX CTRL *vs* LERKO SHAM CTRL by two-way ANOVA followed by Bonferroniȏs *post hoc* test. **c** Food intake normalized over body weight (BW) expressed as g/g. Data are shown as mean ± SEM (n = 8). ***p<0.001 and **p<0.005 OVX *vs* SHAM; °p<0.05 LERKO OVX CTRL *vs* ERα$^{f/f}$ OVX CTRL by two-way ANOVA followed by Bonferroniȏs *post hoc* test. **d** Feeding efficiency expressed as ΔBW/ΔFI (ΔBody weight/ΔFood intake). Data are represented as mean ± SEM (n = 8). *p<0.05 and ***p<0.001 OVX *vs* SHAM; °p<0.05 LERKO OVX *AA *vs* ERα$^{f/f}$ OVX *AA and °°°p<0.001 LERKO OVX CTRL *vs* ERα$^{f/f}$ OVX CTRL by two-way ANOVA followed by Bonferroniȏs *post hoc* test. Source data are provided as a Source Data file.

Supplementary Fig. 6

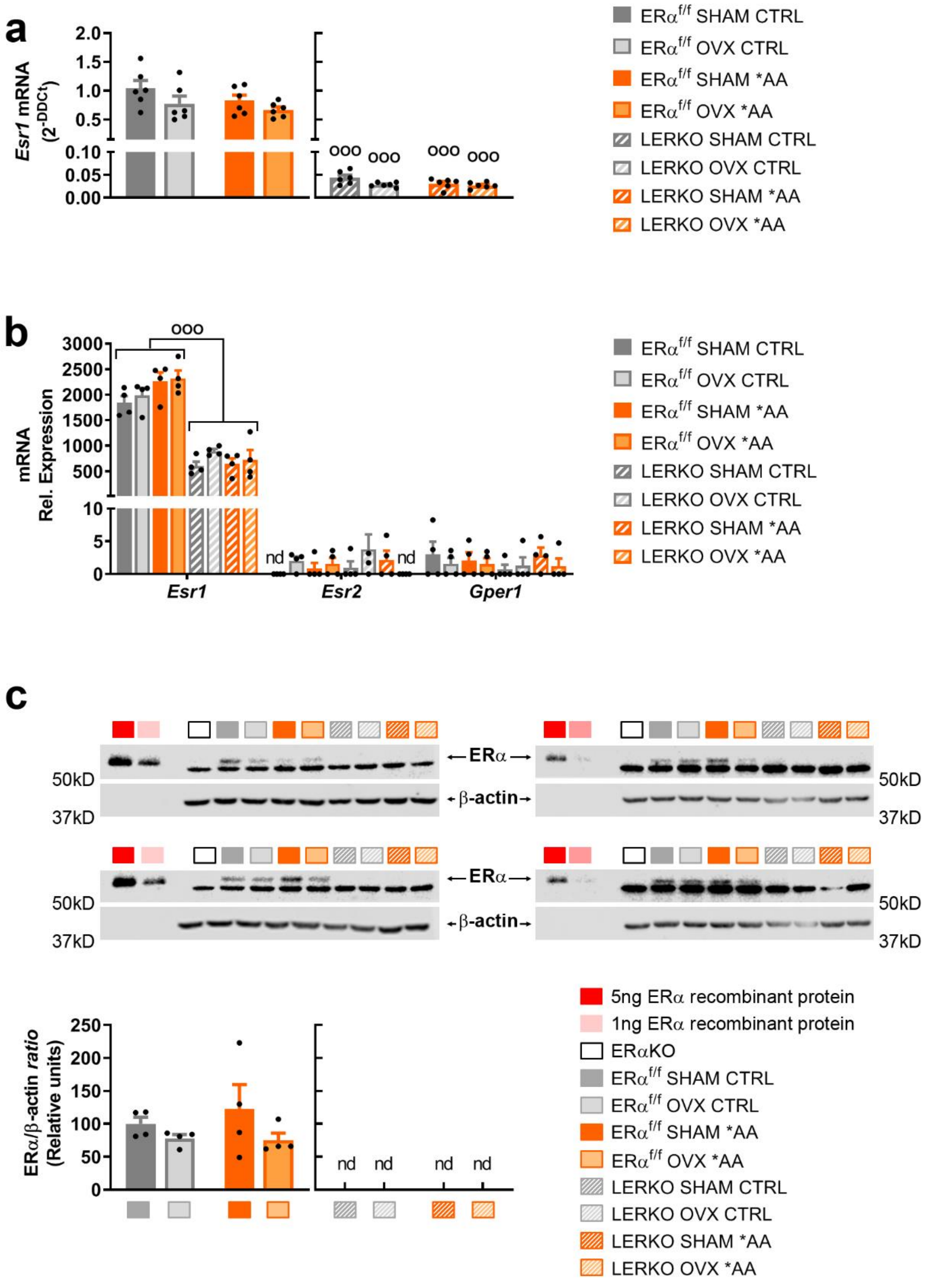

Supplementary Fig. 6. Dietary treatment did not affect the hepatic expression of ERs. a mRNA content of *Esr1* (codifying for ERŬ) measured by RTPCR in the liver of SHAM and OVX ERα$^{f/f}$ and LERKO females fed with CTRL or *AA diet. Data are represented as mean ± SEM (n=6). °°°p<0.001 LERKO *vs* ERα$^{f/f}$ by two-way ANOVA followed by Bonferroniĝs *post hoc* test.

b Relative expression of mRNA content of *Esr1* (codifying for ERŬ), *Esr2* (codifying for ERɓ), and *Gper1* (codifying for GPR30) measured by RNA-Seq in the liver of SHAM and OVX ERα$^{f/f}$ and LERKO females fed with CTRL or *AA diet. Data are mean ± SEM (n=4). °°°*p*<0.001 LERKO *vs* ERα$^{f/f}$ by two-way ANOVA followed by Bonferroniφs *post hoc* test. **c** Representative western blot and semiquantitative analysis of ERŬ protein in liver extracts from SHAM and OVX ERα$^{f/f}$ and LERKO females fed with CTRL or *AA diet. ERŬ recombinant protein and liver extract from ERŬKO mouse were used as positive and negative control, respectively. Data are represented as mean ± SEM (n=4). Uncropped and unprocessed scans of the blots and source data are provided as a Source Data file.

Supplementary Fig. 7

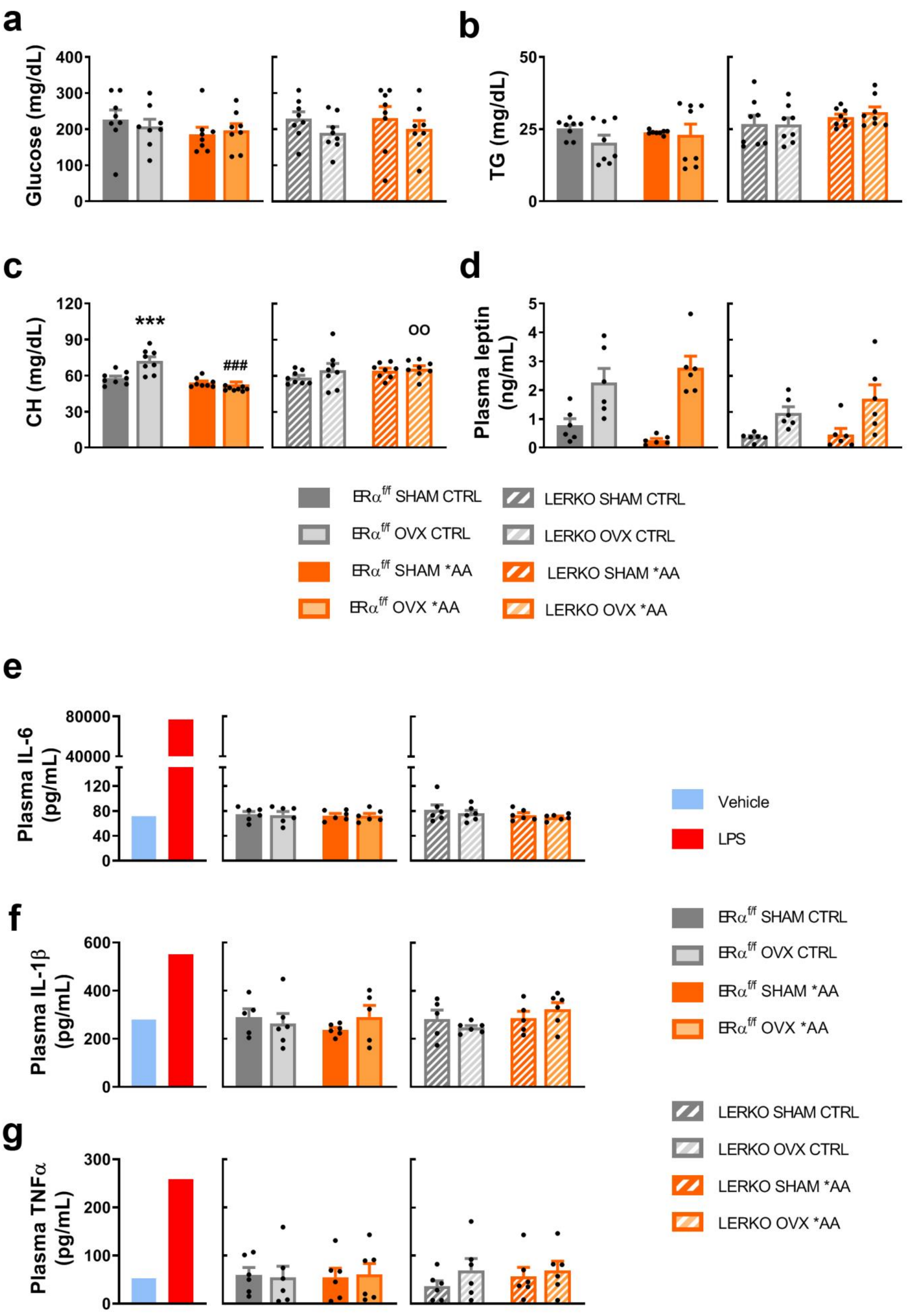

Supplementary Fig. 7. Circulating levels of metabolic and inflammatory markers. a-d Circulating levels of glucose (**a**), triglycerides (TG) (**b**), cholesterol (CH) (**c**), and leptin (**d**) measured in the plasma of SHAM and OVX ERα$^{f/f}$ and LERKO females fed with CTRL or *AA diet. Data are shown as mean ± SEM (n = 8). ****p*<0.001 OVX *vs* SHAM; ###*p*<0.001 *AA *vs* CTRL diet; °°*p*<0.005 LERKO *vs* ERα$^{f/f}$ by two-way ANOVA followed by Bonferroniȏs *post hoc* test. **e-g** Circulating levels of interleukin 6 (IL-6) (**e**), interleukin 1ϐ (IL-ϐ) (**f**) and tumor necrosis factor Ŭ (TNFŬ) (**g**) measured in the plasma of SHAM and OVX ERα$^{f/f}$ and LERKO females fed with CTRL or *AA diet. Data are shown as mean ± SEM (n = 6). The levels of circulating cytokines in mice treated with vehicle or 15mg/Kg Lipopolysaccharide (LPS) are shown as internal control of the assays. Source data are provided as a Source Data file.

Supplementary Fig. 8

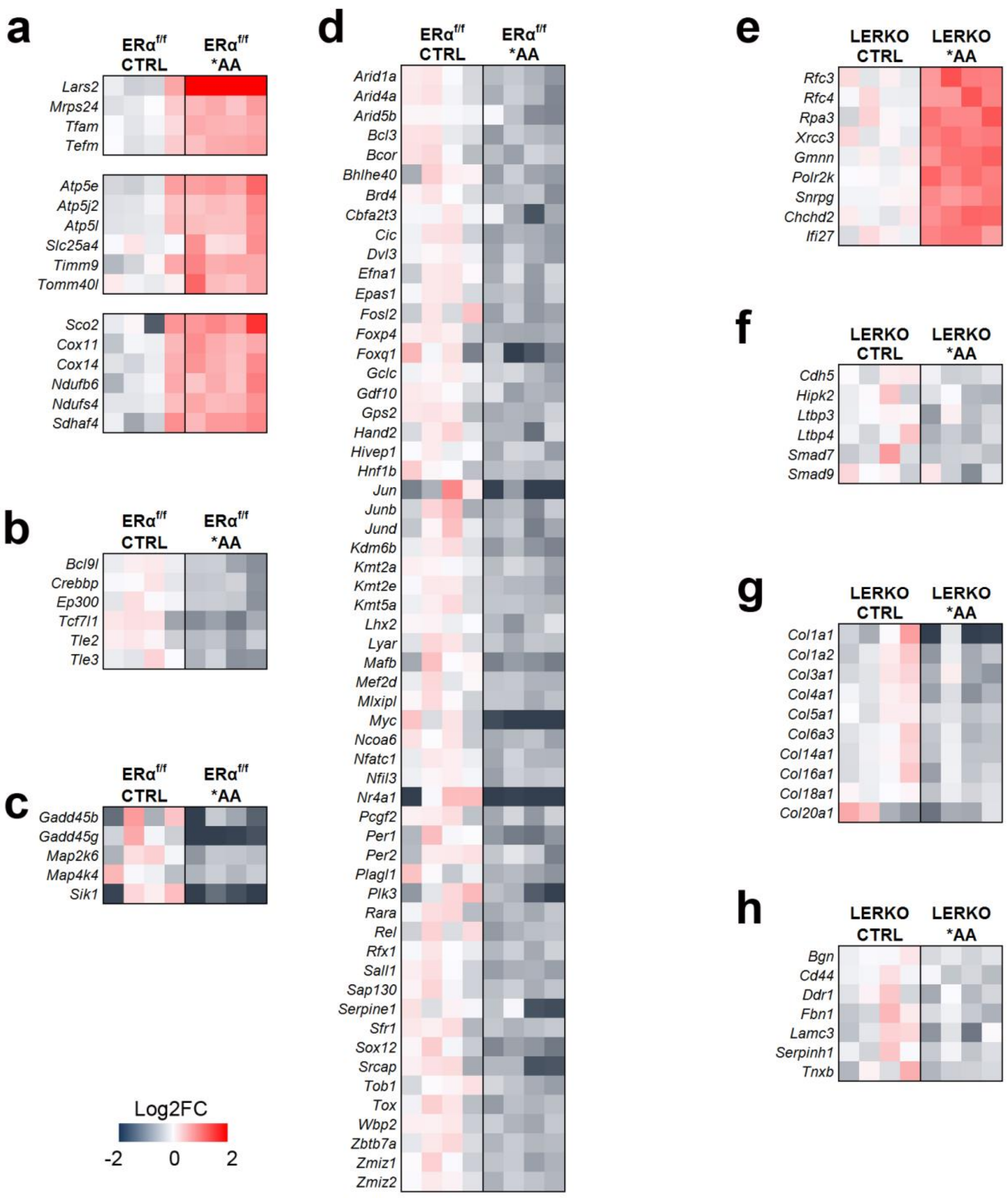

Supplementary Fig. 8. *AA-induced changes in the liver of SHAM females. a-d Heatmaps reporting as Log$_2$FC the expression of genes modulated by *AA diet exclusively in the liver of ERŮ$^{f/f}$ females and associated with ATP synthesis/metabolism and mitochondrial respiratory chain (**a**), ϐ-catenin TCF assembly pathway (**b**), cell cycle (**c**), regulation of transcription (**d**) from RNA-Seq analysis (n=4). **e-h** Heatmaps reporting as Log$_2$FC the expression of genes modulated by *AA diet exclusively in the liver of LERKO females and associated with DNA repair (**e**), TGF-ϐ1 signaling pathway (**f**), collagen metabolism (**g**), extracellular matrix organization (**h**) from RNA-Seq analysis (n=4). Source and processed data are provided in [1] and as a Source Data file.

Supplementary Fig. 9

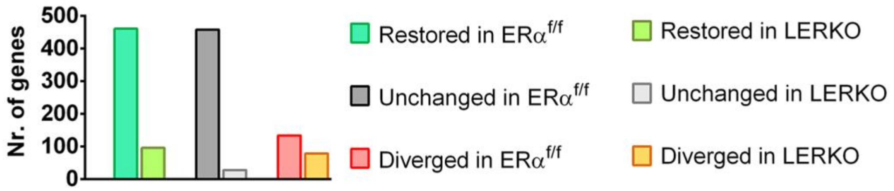

Supplementary Fig. 9. *AA diet-induced changes in the liver of OVX females: comparison between ERŬ$^{f/f}$ and LERKO females. Cluster analysis was performed with Genesis software to identify the number of genes belonging to ñrestoredò, ñunchangedò, and ñdivergedò class in ERα$^{f/f}$ and LERKO females (see also Supplementary Fig. 3). Source and processed data are provided in [1] and as a Source Data file.

Supplementary Fig. 10

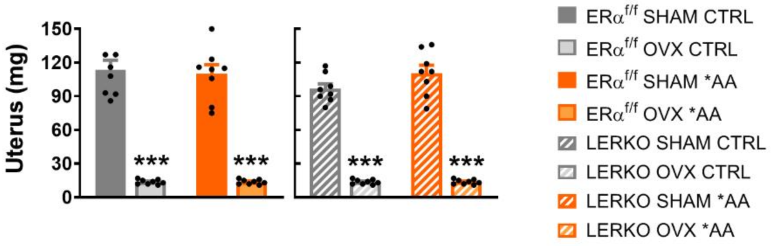

Supplementary Fig. 10. OVX but not *AA diet significantly affects the uterus weight. Uterine weight changes in SHAM and OVX ERα$^{f/f}$ and LERKO females fed with CTRL or *AA diet. Data are shown as mean ± SEM (n = 8). ****p*<0.001 OVX *vs* SHAM by two-way ANOVA followed by Bonferroniȏs *post hoc* test. Source data are provided as a Source Data file.

Supplemental Table 1: Formula of the diets used in the study.

	CTRL (g)	*AA (g)
L-Cystine	3	3
L-Arginine	6	3.6
L-Histidine	4.6	5.8
L-Isoleucine	7.6	16.87
L-Leucine	15.8	34.22
L-Lysine	13.2	20.74
L-Methyionine	5.1	4.1
L-Phenylalanine	8.4	7.1
L-Threonine	7.2	11.3
L-Tryptophan	2.1	1.7
L-Valine	9.3	17.87
L-Alanine	5.1	3.1
L-Cysteine	4.2	5.5
L-Aspartic acid	12.1	7.3
L-Glutamic acid	38.2	23.2
L-Glycine	3	1.8
L-Proline	17.8	10.8
L-Serine	10	6.1
L-Tyrosine	9.2	6.2
Corn Starch	315	315
Maltodextrin 10	35	35
Sucrose	350	350
Cellulose, BW200	50	50
Soybean Oil	25	25
Lard	20	20
Mineral Mix S10026	10	10
Di Calcium Phosphate	13	13
Calcium Carbonate	5.5	5.5
Potassium Citrate, 1 H_2O	16.5	16.5
Vitamin Mix V10001	10	10
Choline Bitartrate	2	2
Dye	0.05	0.05
Kcal/g	3.85	3.85

Supplemental Table 2: Sequence of the primers used for RT-PCR analysis.

Gene	Primer sequence	Source	Code
Esr1	-	ThermoFisher	Mm00433147_m1
36b4	For: 5ô-GGCGACCTGGAAGTCCAACT-3ô	Suppl. Reference [2]	-
36b4	Rev: 5ô-CCATCAGCACCACAGCCTTC-3ô	Suppl. Reference [2]	-

Supplementary Methods

Real-Time PCR Gene Expression Analysis

Total liver RNA extraction was isolated with TRIzol Reagent (Invitrogen, #15596026) and purified using the RNeasy minikit protocol (Qiagen, #74034), according to the manufacturerǿs instructions. For the preparation of cDNA, 1 εg RNA was denatured at 75°C for 5 min in the presence of 1.5 εg of random primers (Promega, #C1181) in 15 εL final volume. Deoxynucleotide triphosphate (GE Healthcare, #GEH28406552) and Moloney murine leukemia virus reverse transcriptase (M-MLV RT) (Promega, #M3682) were added at 0.5 mM and 8 U/εL final concentration, respectively, in a final volume of 25 εL. The RT reaction was performed at 37°C for 1 h; the enzyme was inactivated at 75°C for 5 min. Control reactions without addition of the RT enzyme were performed for each sample. For the real-time PCR experiments, the reaction mix for each sample was made up of 2 εL of pre-diluted cDNA, 5 εL of TaqMan 2× Universal PCR Master Mix No AmpErase UNG (ThermoFisher/Life Technologies, #4324018), 0.5 εL of 20x primers/probes mix, and 2.5 εL of H_2O. The primers used for the Real-Time PCR reactions were listed in Supplementary Table 2. The 36b4 primer was used as reference gene assay. The reaction was carried out according to the manufacturerǿs protocol using QuantStudioÊ 3 Real-Time PCR System with the following thermal profile: 2 min at 50°C; 10 min 95°C; 40 cycles (15 sec 95°C, 1 min at 60°C), and data were analyzed using the 2ī ^{φφCt} method[3].

Biochemical Assays

The levels of glucose were measured with a glucometer according to the manufacturerǿs protocols. The levels of TG (BVN-K622-100, Biovision) and CH (BVN-K603-100, Biovision) in the plasma were measured with appropriate kits according to the manufacturerǿs protocols.

Mouse Leptin ELISA assay

Plasma leptin content was measured with the EZML-82k kit (Millipore) according to the manufacturerǿs protocols.

Cytokines levels

The levels of IL-6, IL-1ϐ and TNFŬ were measured with ELISA Duoset kits (#DY406, #DY401 and #DY410, all from R&D System) according to the manufacturer's instructions. Plasma from mice treated i.p. with vehicle (0.9% NaCl) or 15mg/Kg of LPS for 6 hs were used as internal control of the assays.

Western blot analysis

Samples of frozen mouse liver were homogenized in ice-cold buffer (20 mM HEPES, 5 mM MgCl2, 420 mM NaCl, 0.1 mM EDTA, and 20% glycerol) containing protease and phosphatase inhibitors (Phosphatase and Protease Inhibitor Mini Tablets, Pierce). After 3 repeated cycles of freezing and thawing, the homogenate was centrifuged at 16100g for 15 min at 4°C, and the supernatant was collected in a new tube. After appropriate quantitative analysis (Bradford assay, Pierce), equal amounts of the protein samples (50 µg of liver extracts) were resuspended in Laemmli sample buffer and separated in an 10% SDS polyacrylamide gel. 1ng and 5ng of ERŬ recombinant protein (P2187, Panvera) and 50 µg of liver extract from ERŬKO mouse were used as positive and negative control, respectively. After transfer and blocking in 5% non-fat dried milk diluted in TBST (TBS, 0.1% Tween® 20), the nitrocellulose membranes were cut in correspondence of 50kD; the upper and lower part were incubated overnight at 4°C with the anti-ERŬ and anti-β-actin antibody, respectively. Then, membranes were incubated with the specific secondary antibody conjugated with peroxidase for 1h at r.t. The primary antibodies used were the following: anti-ERŬ (from Thermo Scientific; MA5-14598, dilution 1:1000) and anti-β-actin (from Sigma; A1978, dilution 1:4000). The secondary antibodies used were the following: goat anti-rat IgG (from Sigma-MERCK; AP136P, dilution 1:2000) and horse anti-mouse IgG (from VECTOR Laboratories; PI-2000, dilution 1:2000). Immunoreactivity was detected with an ECL Western Blotting Analysis System (Amersham) and acquired and analyzed using an Odissey Fc Imaging system and the Image StudioÊ software (LiCorBiosciences). Uncropped and unprocessed scans of the blots are provided as a Source Data file.

Supplementary References

1. Della Torre, Sara. RNA-Seq of mouse liver female transcriptome: role of estrogens and hepatic ERa in the metabolic adaptation to dietary BCAAs. (2021). https://www.ncbi.nlm.nih.gov/bioproject/PRJNA778593.
2. Villa, A. *et al.* Tetradian oscillation of estrogen receptor is necessary to prevent liver lipid deposition. *Proc. Natl. Acad. Sci.* **109**, 11806ï 11811 (2012).
3. Schmittgen, T. D. & Livak, K. J. Analyzing real-time PCR data by the comparative CT method. *Nat. Protoc.* **3**, 1101ï 1108 (2008).
