# Supplementary material for: Dietary essential amino acids restore liver metabolism in ovariectomized mice via hepatic estrogen receptor α
Source: Nat Commun. 2021 Nov 25;12:6883. doi: 10.1038/s41467-021-27272-x (PMC8617046; doi:10.1038/s41467-021-27272-x)
Supplement: Supplementary file 2 — Reporting Summary [file 41467_2021_27272_MOESM2_ESM.pdf]

nature research

Corresponding author(s): Sara Della Torre and Adriana Maggi

Last updated by author(s): Jul 19, 2021

Reporting Summary

Nature Research wishes to improve the reproducibility of the work that we publish. This form provides structure for consistency and transparency in reporting. For further information on Nature Research policies, see our Editorial Policies and the Editorial Policy Checklist.

Statistics

For all statistical analyses, confirm that the following items are present in the figure legend, table legend, main text, or Methods section.

n/a	Confirmed	
☐	☒	The exact sample size (*n*) for each experimental group/condition, given as a discrete number and unit of measurement
☐	☒	A statement on whether measurements were taken from distinct samples or whether the same sample was measured repeatedly
☐	☒	The statistical test(s) used AND whether they are one- or two-sided<br>*Only common tests should be described solely by name; describe more complex techniques in the Methods section.*
☒	☐	A description of all covariates tested
☐	☒	A description of any assumptions or corrections, such as tests of normality and adjustment for multiple comparisons
☐	☒	A full description of the statistical parameters including central tendency (e.g. means) or other basic estimates (e.g. regression coefficient) AND variation (e.g. standard deviation) or associated estimates of uncertainty (e.g. confidence intervals)
☐	☒	For null hypothesis testing, the test statistic (e.g. *F*, *t*, *r*) with confidence intervals, effect sizes, degrees of freedom and *P* value noted<br>*Give P values as exact values whenever suitable.*
☒	☐	For Bayesian analysis, information on the choice of priors and Markov chain Monte Carlo settings
☒	☐	For hierarchical and complex designs, identification of the appropriate level for tests and full reporting of outcomes
☒	☐	Estimates of effect sizes (e.g. Cohen's *d*, Pearson's *r*), indicating how they were calculated

Our web collection on statistics for biologists contains articles on many of the points above.

Software and code

Policy information about availability of computer code

Data collection	No software was used for data collection
Data analysis	FASTQC (v0.11.5), STAR aligner (v2.5.2a), Samtools (v1.1), DESeq2 (v1.30.0), Bioinformatics & Evolutionary Genomics software (http://bioinformatics.psb.ugent.be/webtools/Venn/), Cytoscape (v3.7.1), David (v6.8), Enrichr (https://maayanlab.cloud/Enrichr/), REVIGO (http://revigo.irb.hr/), Genesis (v1.8.1), and Shinyheatmap (http://shinyheatmap.com/).

For manuscripts utilizing custom algorithms or software that are central to the research but not yet described in published literature, software must be made available to editors and reviewers. We strongly encourage code deposition in a community repository (e.g. GitHub). See the Nature Research guidelines for submitting code & software for further information.

Data

Policy information about availability of data

All manuscripts must include a data availability statement. This statement should provide the following information, where applicable:

- Accession codes, unique identifiers, or web links for publicly available datasets
- A list of figures that have associated raw data
- A description of any restrictions on data availability

Mouse reference genome GRCm38 is available at the web page https://www.ncbi.nlm.nih.gov/grc/mouse. Gencode GRCm38 primary assembly M20 and the associated GTF annotation file are available at the web page https://www.gencodegenes.org/mouse/release_M20.html.
The RNA-Seq raw data generated in this study have been deposited in the BioProject database under accession code ph4b9 (doi:10.17605/OSF.IO/PH4B9). The RNA-Seq raw data are available after publication acceptance. The processed RNA-Seq data generated in this study are provided in the Source Data file.
Source data are provided with this paper.

Field-specific reporting

Please select the one below that is the best fit for your research. If you are not sure, read the appropriate sections before making your selection.

☒ Life sciences ☐ Behavioural & social sciences ☐ Ecological, evolutionary & environmental sciences

For a reference copy of the document with all sections, see nature.com/documents/nr-reporting-summary-flat.pdf

Life sciences study design

All studies must disclose on these points even when the disclosure is negative.

Sample size	The sample size in this study is equal to 8 (n=8). Sample size was estimated by using the software G*Power 3 (http://www.3rs-reduction.co.uk/html/6__power_and_sample_size.html) and the following parameters derived from our previous studies: 5% significance level, 1.8 S/N ratio, 90% power.
Data exclusions	No data were excluded from the analysis
Replication	The experiment was repeated twice. Both attempts were successful.
Randomization	The samples used for the various analyses were selected a priori, after having randomized the mice of each experimental group.
Blinding	In our study blinding was not applicable during data collection, since we needed to distinguish, for example, mice fed with 2 different type of diets and we need to distinguish SHAM vs OVX females to collect SHAM females in a specific phase of the estrous cycle as stated in the M&M section. Data analysis (i.e. RNA-Seq analysis) was performed in a blind fashion.

Reporting for specific materials, systems and methods

We require information from authors about some types of materials, experimental systems and methods used in many studies. Here, indicate whether each material, system or method listed is relevant to your study. If you are not sure if a list item applies to your research, read the appropriate section before selecting a response.

Materials & experimental systems

n/a	Involved in the study	
☐	☒	Antibodies
☒	☐	Eukaryotic cell lines
☒	☐	Palaeontology and archaeology
☐	☒	Animals and other organisms
☒	☐	Human research participants
☒	☐	Clinical data
☒	☐	Dual use research of concern

Methods

n/a	Involved in the study	
☒	☐	ChIP-seq
☒	☐	Flow cytometry
☒	☐	MRI-based neuroimaging

Antibodies

Antibodies used	The primary antibodies used were the following: anti-ERα (from Thermo Scientific; MA5-14598, dilution 1:1000) and anti-β-actin (from Sigma; A-1978, dilution 1:4000). The HRP-conjugated secondary antibodies used were the following: goat anti-rat IgG (from Sigma-MERCK; AP136P, dilution 1:2000) and horse anti-mouse IgG (from VECTOR Laoratories; PI-2000, dilution 1:2000).
Validation	All the primary antibodies used were validated: by manufactures' web pages (for ERa: https://www.thermofisher.com/de/de/home/technical-resources/research-tools/image-gallery/image-gallery-detail.78864.html; for b-actin: https://www.sigmaaldrich.com/IT/it/product/sigma/a1978?context=product); by previous publications (for b-actin: PMID: 31257151, PMID28604676); by this study itself where 1ng and 5ng of ERα recombinant protein (P2187, Panvera) and 50 µg of liver extract from ERαKO mouse (PMID: 10976058) were used as positive and negative control for ERa, respectively.

Animals and other organisms

Policy information about studies involving animals; ARRIVE guidelines recommended for reporting animal research

Laboratory animals	Transgenic mice: ERaf/f female mice, strain C57BL/6J, 6 months of age at collection time; LERKO female mice, strain C57BL/6J, 6 months of age at collection time.
Wild animals	The study did not involve wild animals.
Field-collected samples	The study did not involve samples collected from the field.

Ethics oversight | All animal experimentation was done in accordance with the ARRIVE and European guidelines for animal care and use of experimental animals. The study was approved by the Italian Ministry of Research and University and a Departmental panel of experts was responsible for the control of all handling and surgical protocols.

Note that full information on the approval of the study protocol must also be provided in the manuscript.
